# “γδT Cell-IL17A-Neutrophil” Axis Drives Immunosuppression and Confers Breast Cancer Resistance to High-Dose Anti-VEGFR2 Therapy

**DOI:** 10.3389/fimmu.2021.699478

**Published:** 2021-10-15

**Authors:** Zhigang Zhang, Chenghui Yang, Lili Li, Ying Zhu, Ke Su, Lingyun Zhai, Zhen Wang, Jian Huang

**Affiliations:** ^1^ Department of Gynecology, The Second Affiliated Hospital, Zhejiang University School of Medicine, Zhejiang University, Hangzhou, China; ^2^ Key Laboratory of Tumor Microenvironment and Immune Therapy of Zhejiang Province, The Second Affiliated Hospital, Zhejiang University School of Medicine, Hangzhou, China; ^3^ Department of Breast Surgery, The First Affiliated Hospital, Wenzhou Medical University, Wenzhou, China; ^4^ Department of Medical Oncology, The Second Affiliated Hospital, Zhejiang University School of Medicine, Hangzhou, China; ^5^ Department of Breast Surgery, The Second Affiliated Hospital, Zhejiang University School of Medicine, Hangzhou, China; ^6^ Cancer Center, Zhejiang University, Hangzhou, China

**Keywords:** γδT cell, neutrophil, anti-VEGFR2 therapy, breast cancer, IL17A, therapy resistance

## Abstract

Angiogenesis is an essential physiological process and hallmark of cancer. Currently, antiangiogenic therapy, mostly targeting the vascular endothelial growth factor (VEGF)/VEGFR2 signaling axis, is commonly used in the clinic for solid tumors. However, antiangiogenic therapies for breast cancer patients have produced limited survival benefits since cancer cells rapidly resistant to anti-VEGFR2 therapy. We applied the low-dose and high-dose VEGFR2 mAb or VEGFR2-tyrosine kinase inhibitor (TKI) agents in multiple breast cancer mouse models and found that low-dose VEGFR2 mAb or VEGFR2-TKI achieved good effects in controlling cancer progression, while high-dose treatment was not effective. To further investigate the mechanism involved in regulating the drug resistance, we found that high-dose anti-VEGFR2 treatment elicited IL17A expression in γδ T cells *via* VEGFR1-PI3K-AKT pathway activation and then promoted N2-like neutrophil polarization, thus inducing CD8^+^ T cell exhaustion to shape an immunosuppressive microenvironment. Combining anti-VEGFR2 therapy with immunotherapy such as IL17A, PD-1 or Ly-6G mAb therapy, which targeting the immunomodulatory axis of “γδT17 cells-N2 neutrophils” *in vivo*, showed promising therapeutic effects in breast cancer treatment. This study illustrates the potential mechanism of antiangiogenic therapy resistance in breast cancer and provides synergy treatment for cancer.

## Introduction

Pathological angiogenesis is a key feature of cancer and is involved in multiple stages of cancer development (1). Neovascularization can be enacted by a number of different mechanisms and multiple proangiogenic factors in the tumor microenvironment (such as VEGF and ANG-2) (2). Currently, antiangiogenic therapies, mostly targeting the VEGF/VEGFR2 signaling, are widely applied in the clinic and considered as a major treatment for a wide range of advanced or metastatic tumors, including breast, colon, lung and gynecological cancers (3, 4). Interestingly, the efficacy of antiangiogenic drugs in the treatment of breast cancer is inconsistent. Clinical trials found that antiangiogenic therapy achieved only modest gains in progression-free survival and had no effect on overall survival (5, 6). Thus, the clinical guideline recommendations may not be universally applicable. The relapse mechanisms under antiangiogenic therapy remain unclear.

Antiangiogenic therapy cannot kill tumor cells directly, but works by changing the tumor microenvironment, such as restoring tumor perfusion and proper oxygenation to limit tumor cell invasion. Studies have also revealed that many factors in the tumor microenvironment can influence the effects of antiangiogenic drugs (7–9). Evidence suggests that myeloid cells, the largest population of innate immune cells, are crucial for resistance to antiangiogenic therapies *via* their immunosuppressive pathways (10). Previous studies have reported that neutrophils produce VEGF, MMP9, and Bv8 to sustain tumor angiogenesis (11–13). However, how neutrophils are functionally differentiated during antiangiogenic therapy remains unknown.

γδ T cells are a subtype of innate-like T lymphocytes that perform critical functions in the immune system and are conducive to either the immune response or immune regulation depending on their environment (14, 15). In the tumor microenvironment, γδ T cells are a remarkably heterogeneous population. Their antitumor effects are mediated by direct tumor cell killing *via* secreting interferon (IFN)-γ but they can also secret IL17A to promote tumor growth (16). Coffelt’s research showed that, in a breast cancer mouse model, IL17A-producing γδ T cells induced neutrophil expansion and polarization, which created a premetastatic immunosuppressive microenvironment (17). However, it has not been reported whether γδ T cells are involved in antiangiogenic tumor therapy.

In this study, to clarify the mechanism of resistance to high-dose anti-VEGFR2 therapy, we applied different doses of VEGFR2 mAb and VEGFR2-tyrosine kinase inhibitor (TKI) in various breast cancer mouse models. Our results revealed that γδ T cells and neutrophils were actively involved in tumor resistance to high-dose anti-VEGFR2 therapy. Furthermore, we found that high-dose VEGFR2-TKI induced γδ T cells to produce IL17A *via* VEGFR1-PI3K-AKT pathway activation, which in turns promoted N2-like neutrophil polarization. N2 neutrophils accelerrated the CD8+ T cells exhaustion and then shaped an immunosuppressive microenvironment. Finally, we uncovered the “γδ T17 cells- N2 neutrophils” immunomodulatory axis that instigates resistance to high-dose anti-VEGFR2 therapy.

## Materials and Methods

### Cell Lines

The 4T1 mammary tumor cell line was obtained from the Shanghai Institute of Cell Biology of the Chinese Academy of Science (SIBS, Shanghai, China), and the EMT6 mammary tumor cell lines were obtained from FuDan IBS Cell Center (FDCC, Shanghai, China). Both cell lines were authenticated by STR profiling and incubated in a humidified incubator with 5% CO_2_ at 37°C. They were cultured in RPMI-1640 complete medium supplemented with 10% FBS (Gibco, USA) and 1:100 penicillin-streptomycin (Gibco).

### Mice

Female wild-type BALB/c mice were provided by Slaccas Co. (Shanghai, China), and FVB/N-Tg (MMTV-PyMT) mice were provided by Gempharmatech (Nanjing, China). All mice were housed in the specific pathogen-free conditions of Zhejiang Chinese Medical University Laboratory Animal Research Center. All mouse protocols and procedures were reviewed and approved by the Ethics Review Committee of the Second Affiliated Hospital of the Zhejiang University School of Medicine.

In order to establish the 4T1 or EMT6 tumor-bearing mouse model, BALB/c mice were anesthetized with 0.8% sodium pentobarbital (80 mg/kg) intraperitoneal (*i.p.*) and inoculated with a suspension of 1x10^5^ 4T1 or EMT6 cells in the right fourth mammary fat pad.

### VEGFR2 Tyrosine Kinase Inhibitors (TKI) and VEGFR2 Monoclonal Antibody (mAb) Treatment

**Figure 1:**
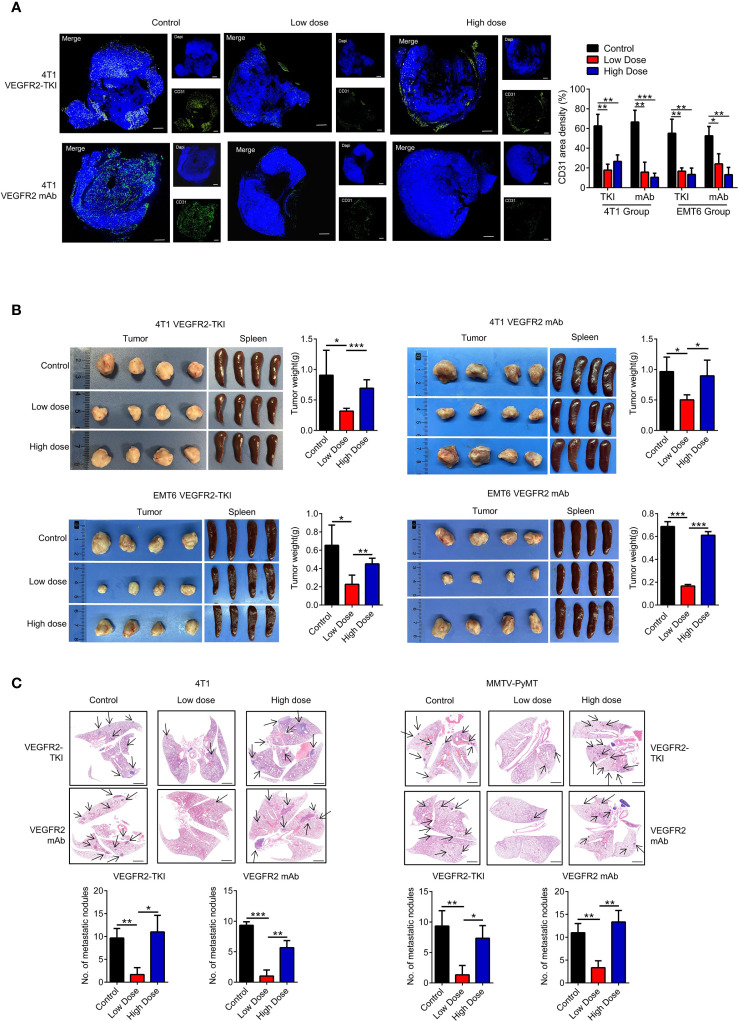
High doses of anti-VEGFR2 therapy are not effective at controlling breast cancer progression. **(A)** Immunofluorescence and quantification of CD31 on breast cancer tissue after anti-VEGFR2 therapy in 4T1 model. Bar=200 μm. Green, CD31; blue, DAPI. **(B)** Representative size and weight of tumor and spleen after anti-VEGFR2 therapy. **(C)** H&E staining and quantification of lung metastasis in the 4T1 model (Black arrow) and MMTV-PyMT model (Black arrow) after anti-VEGFR2 therapy. Bar=1 mm. Data are presented as the means ± SD from one representative experiment. Similar results were obtained from three independent experiments, n=4 mice each group, unless indicated otherwise. Statistical analysis was performed by one-way ANOVA. *p < 0.05, **p < 0.01, and ***p < 0.001. See also **Supplementary Figures S1**, **S2**.

The VEGFR2-TKI YN968D1 (apatinib) that inhibited VEGFR2 was obtained from Jiangsu Hengrui Medicine Co. (Nanjing, China) (18). Anti-mouse VEGFR2 mAb was obtained from BioXCell (clone DC101). VEGFR2-TKI YN968D1 was intragastric (*i.g.*) administrated for mice (low dose: 100mg/kg/day, high dose: 200mg/kg/day) and Anti-mouse VEGFR2 mAb was *i.p.* administrated for mice (low dose: 2mg/kg/twice a week, high dose: 10mg/kg/twice a week). The details of the strategy are shown in **Supplementary Figures S1A**, **S2A**. The primary tumor CD31 expression level, primary tumor size and tumor weight, spleen weight, and numbers of lung metastatic nodules were measured to evaluate the therapeutic effect of VEGFR2-TKI and VEGFR2 mAb (**Figure 1** and **Supplementary Figure S1**).

### Specimen Acquisition and Processing

The spleen was ground on 100-μm nylon mesh (BD Falcon, #352360) into single cells. The splenocyte suspensions were lysed for 1 min (BD Bioscience, #349202) before subsequent detection or culture. Bone marrow cells were obtained from the hind limbs and then filtered into single cells. Primary tumors were cut into small pieces after excluding connective tissue and digested in digestion medium containing RPMI-1640 with 1 mg/ml collagenase IV (Sigma-Aldrich, #V900893) in a 37°C shaker for 1-2 h until digestion was complete. The single-cell suspension was further filtered through 40-μm nylon mesh (BD Falcon, #352340) to effectively remove impurities.

### Flow Cytometry Analysis and Sorting

For cell surface marker staining, primary tumor cells, bone marrow cells and splenocytes were isolated as described previously (19) and incubated with Zombie Red™ Fixable Viability Kit (Biolegend, #423109, to assess live vs. dead status of cells) for 30 min at room temperature (RT) and then washed with PBS. Cell suspension was then stained with anti-CD16/32 (clone S17011E, to block non-specific binding of immunoglobulin to the Fc receptors) for 10 min and then with the following fluorochrome-conjugated mAbs for 30 min at 4°C: anti-CD45 (clone 30-F11), anti-CD3ϵ (clone 145-2C11), anti-CD4 (clone RM4-5), anti-CD8a (clone 53-6.7), anti-TCRγ/δ (clone GL3), anti-TCRβ (clone H57-597), anti-CD11b (clone M1/70), anti-Ly6G (clone 1A8), anti-EpCAM (clone G8.8), anti-PD-1 (clone RMP1-30), anti-PD-L1 (clone 10F.9G2), anti-VEGFR2 (clone 89B3A5), anti-CD14 (clone Sa14-2), anti-Ly6C (clone HK1.4) (all from BioLegend) and anti-VEGFR1/Flt-1 (clone 141522), anti-VEGFR3/Flt-4 (Polyclonal) (from R&D Systems). Isotype controls are applied as negative controls.

For intracellular staining, the cell density was adjusted to 5×10^6^/ml, and a cell stimulation cocktail plus protein transport inhibitors (eBioscience, #00-4975-03) was added to the plate and incubated for 4-6 h. Cell surface marker staining was performed as described above, and the cells were then fixed and permeabilized using a fixation buffer (BioLegend, #420801) and permeabilization wash buffer (BioLegend, #421002). Subsequently, the cells were stained with fluorophore-conjugated antibodies against IFN-γ (clone XMG1.2, BioLegend) and IL17A (clone TC11-18H10.1, BioLegend).

For detecting the ROS level of indicated neutrophils, cells were harvested and Reactive Oxygen Species Assay Kit (Beyotime, #S0033) was used for staining.

Data were obtained from a FACSCanto II flow cytometer (BD Biosciences, San Jose, CA) and analyzed with FlowJo software (V10, Tree Star Inc., Ashland, OR). For flow cytometry sorting, a single-cell suspension was sorted with a FACSAria II cell sorter (BD Bioscience). The cell sorting strategy was as follows: ① CD45-APC/Cy7, CD3ϵ-PerCP/Cy5.5, and TCRγ/δ-APC; ② CD45-APC/Cy7, CD11b-PE/Cy7, and Ly-6G-APC.

### Mouse Neutrophil Magnetic Isolation

Mouse neutrophil MACS isolation was performed using a Mouse Neutrophil Isolation Kit (Miltenyi, #130-097-658). Briefly, single-cell suspensions from mice bone marrow or tumor tissue were acquired, and erythrocytes were lysed before magnetic labeling. Then, 50 μl of Neutrophil Biotin-Antibody Cocktail was added per 200 μL of cell suspension (5x10^7^ total cells) and incubated for 15 min on ice. After washing, 100 µl of Anti-Biotin MicroBeads was added per 400 μl of cell suspension. An LS column and a MidiMACS separator (Miltenyi) were used for subsequent magnetic sorting.

### Biochemical Characterization Detection of Neutrophil

Arginase1, Prostaglandin E2 (PGE2), nitric oxide (NO) levels were measured to evaluate the indicated neutrophils’ cellular immunosuppressive function. Arginase Activity Assay Kit (Sigma-Aldrich, #MAK112), Prostaglandin E2 Assay (R&D Systems, #KGE004B), Nitric Oxide Assay Kit (Beyotime, #S0021) were applied. The procedures were performed according to the manufacturers’ protocol.

### Tissue Culture Supernatant Collection

Primary tumor tissue was cut into small pieces using sterile ophthalmic scissors. Then, the samples were placed in a 6-well plate with RPMI-1640 medium. The culture supernatant was harvested after 24 h and centrifuged at 300xg for 5 min for further purification.

### Neutrophil and γδ T Cells Induction *In Vitro*

Mouse bone marrow-derived neutrophils were obtained as previously described. Neutrophils were plated in plate bottom 96-well plates with different concentration of VEGFR2-TKI, tissue culture supernatant, IL17A mAb (1μg/mL, Biolegend, #506945), IFN-γ mAb (1μg/mL, Biolegend, #505833) and cells were harvested after 4h. The neutrophils cellular marker staining procedure is described above. For co-culture assay (**Figures 4D, E**), tumor-infiltrating γδ T cells were obtained from primary tumors *via* single-cell FACS sorting. γδ T cells and neutrophils (cell ratio 1:1) suspended in complete RPMI-1640 medium containing Ultra-LEAF-purified anti-mouse CD3ϵ mAb (BioLegend, #100340) were plated in U-bottom 96-well plates separated with 0.4μm transwell chamber (Corning, #3381).

For γδ T induction *in vitro* (**Figures 3B, D** and **Supplementary Figure S3G**), mouse spleen-derived γδ T cells were obtained by MACS isolation. γδ T cells suspended in complete RPMI-1640 medium containing purified anti-mouse CD3ϵ mAb were plated in U-bottom 96-well plates and different concentration of VEGFR2-TKI (**Figures 3B, D**) and YS-49 (**Supplementary Figure S3G**).

### CD8^+^ T Cell Detection and Co-Culture System *In Vitro*

CD8^+^ T cells were isolated from naive BALB/c mouse spleens using a positive CD8^+^ T cell isolation kit (BioLegend, #100704). For T cell proliferation assays, MACS-isolated CD8^+^ T cells were firstly labeled with CFSE (1 μM, BioLegend, #423801) in a 37°C cell culture incubator for 10 min and thoroughly washed 3 times with pre-warmed complete RPMI-1640 medium.

For exploring CD8^+^ T cells proliferation (**Figures 6A**, **Supplementary Figures S6B, D**) and PD-1 expression (**Figures 6B**, **Supplementary Figures S6C, E**), cells were suspended in complete RPMI-1640 medium with Ultra-LEAF-purified anti-mouse CD3ϵ mAb, anti-mouse CD28 mAb (BioLegend, #102115) and recombinant IL-2 (PeproTech, #210-12). For detecting CD8^+^ T cells change under the VEGFR2-TKI intervention *in vitro*, different dose VEGFR2-TKI was added in the culture system for 3 days and evaluated *via* flow cytometry. For detecting CD8^+^ T cells change under the influence of tumor-infiltrating neutrophils or γδ T cells, tumor-infiltrating neutrophils or γδ T cells were obtained from primary tumors (low dose VEGFR2-TKI treated, high does VEGFR2-TKI treated or untreated mice) *via* single-cell FACS sorting. γδ T cells or neutrophils were co-cultured with CD8^+^ T cells (cell ratio 10:1) for 3 days and evaluated *via* flow cytometry.

For investigating the γδ T-neutrophil-CD8^+^ T cells axis and the effect of IL17A, The schedule was operated and showed in **Figure 6C**. Briefly, tumor-infiltrating γδ T cells from high does VEGFR2-TKI treated mice primary tumors were plated into upper chamber (0.4μm transwell chamber) and CD8^+^ T cells with or without naive neutrophils were plated into the lower chamber with complete RPMI-1640 medium containing Ultra-LEAF-purified anti-mouse CD3ϵ mAb, anti-mouse CD28 mAb and recombinant IL-2 (γδ T cells: neutrophil: CD8^+^ T cells ratio =10:10:1). IgG control (1μg/mL, Biolegend, #400431) or anti-IL17A mAb (1μg/mL) was added in the coculture system.

### PI3K Activator and Inhibitor Treatment

In order to evaluate the PI3K pathway in tumor development, BALB/c mice were inoculated 4T1 cells in the right fourth mammary fat pad and divided into 5 groups as shown in **Supplementary Figure S3F**: 1. PBS group, 2. low-dose VEGFR2-TKI group, 3. low-dose VEGFR2-TKI with PI3K inhibitor Copanlisib (MCE, #HY-15346, intravenous (*i.v.*) 100 μg/mouse/every other days) group, 4. high-dose VEGFR2-TKI group, 5. high-dose VEGFR2-TKI with PI3K activator YS-49 (MCE, #HY-15346, *i.p.* 100 μg/mouse/every other days) group. Intervention was started two weeks after 4T1 cells inoculation.

### Therapeutic mAb Treatment

Two weeks after 4T1 cell inoculation in the right fourth mammary fat pad, the combination treatment of high-dose VEGFR2-TKI and a mAb was performed for 2 weeks. Anti-IL17A mAb (clone 17F3), anti-Ly-6G mAb (clone 1A8), anti-PD-1 mAb (clone J43), mouse IgG1 (clone MOPC-21) (All from BioXCell, i.v., 100 μg/mouse/every three days) or PBS as control was applied. The mAb deleting efficiency was proved by obtain PB at the end of the treatment and stained with anti-CD45, anti-CD11b, anti-Ly6G *via* flow cytometry.

### Western Blot

Sorted γδ T cells from primary tumor or spleen-derived γδ T cells after *in vitro* treatment were harvested and lysed in pre-cooled RIPA Lysis Buffer (Beyotime, #P0013B) with a cocktail of protease and phosphatase inhibitor (Thermo Fisher, #78445). A bicinchoninic acid (BCA) assay kit (Thermo Fisher, #23227) was used for protein concentration measurement. The proteins were separated by sodium dodecyl sulfatepolyacrylamide gel electrophoresis (SDS-PAGE) and then transferred onto a polyvinylidene difluoride (PVDF) membrane (Bio-Rad). After blocking with 5% (w/v) fat-free milk (BD Biosciences, #232100) at RT for 1 h, the membrane was incubated with the corresponding primary antibodies overnight at 4°C followed by the appropriate horseradish peroxidase (HRP)-conjugated secondary antibodies. Immunoreactive bands were identified using enhanced chemiluminescence (Thermo Fisher Pierce, #32109). Primary antibody, including anti-AKT (1:1000, Cell Signaling Technology, #4685S), anti-Phospho-Akt (Ser473, 1:1000, Cell Signaling Technology, #4058S), anti-PI3 Kinase (p85, 1:1000, Cell Signaling Technology, #4257S), anti-PI3 Kinase p85 (Tyr458)/p55 (Tyr199) (1:1000, Cell Signaling Technology, #4228S), and anti-β-actin (1:2000, HuaBio, #EM21002), was applied. Secondary antibodies, including anti-mouse (1:5000, HuaBio, #G1006-1) and anti-rabbit (1:5000, HuaBio, #HA1001), were applied. Quantification of WB images was conducted by ImageJ software (version 1.48).

### Tissue Immunofluorescence Staining

Mouse tissue was obtained, fixed with 4% paraformaldehyde for 24 h and then embedded in paraffin for sectioning. After dewaxing and dehydration, sections were incubated with a primary antibody overnight. A fluorophore-labeled secondary antibody was added and incubated for 2 h at RT. Finally, the sections were stained with DAPI and imaged. Primary Abs specific for CD31 (Polyclonal, Proteintech, #28083-1-AP) were applied. CD31 area density (%) was calculated by dividing the CD31-positive staining area with the total total tissue area. Quantification of immunofluorescence images was conducted by ImageJ software.

### Giemsa Staining

Collected neutrophils were adjusted to a cell concentration of 1x10^6^/mL. Then, 50 μL of cell suspension was added to a cytospin, and the cells were attached to the slide. Then, the cells were fixed for 20 min in 4% paraformaldehyde, stained with a Giemsa solution (Solarbio, 0.4 w/v, #G1015) for 10 min and washed with ddH_2_O. Cell nuclear morphology was observed under an optical light microscopy by two independent researchers (Chenghui Yang AND Ke Su). 5 fields of view are randomly selected for each sample and cells with nucleus segmentation ≥3 are deemed as N1 type neutrophil while cells with round nucleus are deemed as N2 type neutrophil.

### Statistical Analysis

All statistical analyses were performed using GraphPad Prism (V6.0, GraphPad Software, Inc. La Jolla, CA). The results are expressed as the mean values ± SD. The significance of variations between two groups was determined by an unpaired two-tailed Student’s t test, and one-way ANOVA was also used for the multiple groups comparisons. Repeated-Measures ANOVA was performed for changes over time in the groups. Statistical significance was assumed at *p*<0.05. The following symbols were applied in figures: *p<0.05, **p<0.01, and ***p<0.001.

## Results

### High-Dose Anti-VEGFR2 Therapy Is Not Effective at Controlling Breast Cancer Progression

Previous studies suggested that anti-VEGFR2 treatment inhibit angiogenesis and tumor growth in dose-dependent pattern (20, 21). In order to investigate the changes in intratumoral vessels and tumor through the treatment, we treated the breast cancer mice with administration of an anti-VEGFR2 antibody (clone DC101) or YN968D1 (Apatinib), a small molecule tyrosine kinase inhibitor (TKI) that selectively inhibits VEGFR2.

According to previous studies, 50 to 200mg/kg/day of VEGFR2-TKI (YN968D1) has an anti-tumor effect in different tumor mouse models (18). Combined with the sensitivity of breast cancer to antiangiogenic therapy and related mouse studies, we selected 200mg/kg/day as the high-dose group, and 100mg/kg/day was the relatively low-dose control group. In addition, the optimal biotherapeutic dose of VEGFR2 mAb (DC101) remains controversial and fluctuates widely among mouse strains and tumor types, with a maximum of 50mg/kg, 3 times a week (22, 23). Studies have found that the treatment efficiency is the highest at 6mg/kg dose of DC101, the highest at 13-16mg/kg dose, and then decreases with the increase of dose and duration (24). Combining the published literatures with our former experiments, we selected 10mg/kg twice-weekly intraperitoneal injection as the high-dose group and 2mg/kg twice-weekly as the low-dose control group.

Various breast cancer mice models were conducted in our experiments. We used the metastatic (4T1) and less invasive (EMT6) mammary cell lines in a syngeneic (Balb/c) mouse xenograft model. Murine 4T1 cells were originally derived from a spontaneous breast cancer in the Balb/c strain and have been reported as metastatic, which are largely similar to human basal/triple-negative breast cancer (TNBC) cell lines. Conversely, the EMT6 murine cell lines have been shown to be less invasive and exhibit the characteristics of human Luminal breast cancer subtype. To test the efficacy of VEGFR2-TKI (YN968D1) and VEGFR2 mAb (DC101) in the treatment of breast cancer with different invasive potentials, we established following treatment groups **(** **Supplementary Figure S1A** **)**. Tumor angiogenesis was effectively reduced with different doses of VEGFR2-TKI and VEGFR2 mAb treatment (**Figure 1A** and **Supplementary Figure S1B**). However, the inhibitory effects of anti-VEGFR2 therapy on tumor growth were evident in the low-dose group but not significant in the high-dose group (**Figures 1B**, **Supplementary Figures S1C, D**). Furthermore, the same VEGFR2 treatment was also given to MMTV-PyMT mouse model (**Supplementary Figure S2A**), a spontaneous breast cancer model, and the low-dose group reduced the primary tumor as well as lung metastasis, while no effect was found in the high-dose group (**Figures 1C** and **Supplementary Figures S2B**). This is consistent with current reports that high-dose anti-VEGFR2 therapy is not effective for breast cancer treatment.

### High-Dose VEGFR2-TKI Therapy Contributes to the Polarization of IL17A-Producing γδ T Cells Rather Than IFN-γ-Producing γδ T Cells

Our study and previous studies found that the onset of VEGFR2-TKI (YN968D1) response and relapse is similar to that observed with VEGFR2 mAb (DC101) (22, 25), supporting the notion that VEGFR2-TKI predominantly blocks the VEGFR2 pathway in breast cancer. Therefore, VEGFR2-TKI was used to explore the resistance mechanism.

**Figure 2:**
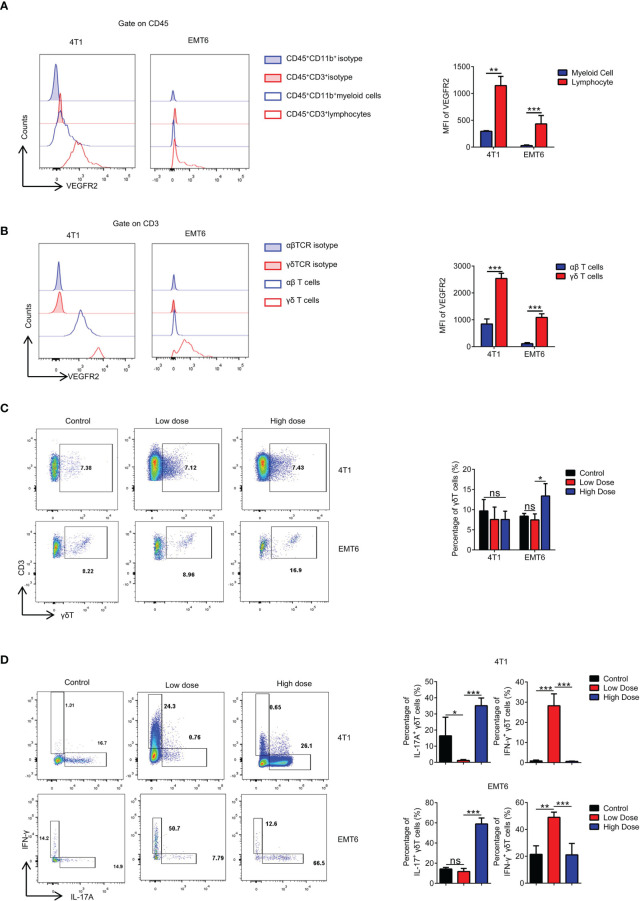
High-dose VEGFR2-TKI therapy contributes to differentiation into IL17A-producing γδT cells rather than IFN-γ producing γδT cells. **(A)** Flow cytometry analysis of VEGFR2 expression of myeloid cells (CD45^+^CD11b^+^) and lymphocytes (CD45^+^CD3^+^). **(B)** Flow cytometry analysis of VEGFR2 expression of αβT cells (CD45^+^CD3^+^TCRβ^+^) and γδT cells (CD45^+^CD3^+^TCRγδ^+^). **(C)** Frequency of γδT cells in the tumor after anti-VEGFR2 therapy. **(D)** Intracellular IFN-γ and IL17A expression of γδT cells from tumors *in vivo*. Data are presented as the means ± SD from one representative experiment. Similar results were obtained from three independent experiments, n=4 mice each group, unless indicated otherwise. Statistical analysis was performed by two-tailed unpaired Student’s t test. ns, not significant, *p < 0.05, **p < 0.01, and ***p < 0.001. See also **Supplementary Figure S3**.

While exploring the immunological factors involved in the resistance mechanism of high-dose VEGFR2-TKI on tumor progression, we found that the therapeutic target VEGFR2 was expressed at significantly higher levels in T lymphocytes than in myeloid cells (**Figure 2A** and **Supplementary Figure S2C**), and γδ T cells exhibited higher VEGFR2 expression than αβ T cells **(** **Figure 2B**). We also found that neither low dose nor high dose affected the proportion of γδ T cells infiltrating the tumor in the 4T1 model, while the high-dose group in EMT6 was abundant in infiltrating γδ T cells (**Figure 2C**). Importantly, IFN-γ-producing γδ T cells were predominant with the low-dose treatment, while IL17A-producing γδ T cells were predominant with the high-dose treatment that induced drug resistance (**Figure 2D**). From these results, we speculated that VEGFR2-TKI acts on γδ T cells directly *via* VEGFR-2, thereby affecting γδ T cell polarization.

### High-Dose VEGFR2-TKI Treatment Activates the “VEGFR1-PI3K-AKT” Pathway to Promote Polarization of γδT17 Cells

**Figure 3:**
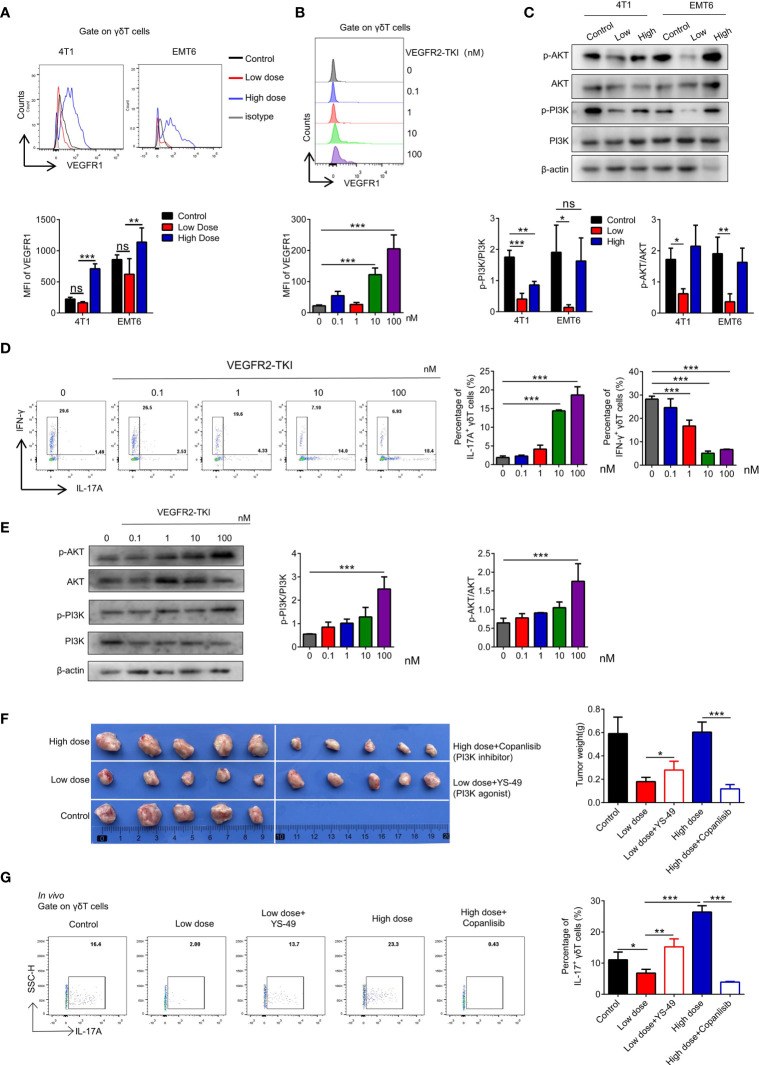
High-dose VEGFR2-TKI therapy induce γδT cells producing IL17A *via* VEGFR1-PI3K-AKT pathway. **(A)** Flow cytometry analysis of VEGFR1 expression of γδT cells (CD45^+^CD3^+^TCRγδ^+^) in the tumor. **(B)** Flow cytometry analysis of VEGFR1 expression of γδT cells derived from naive spleen treated with different doses of VEGFR2-TKI *in vitro*. **(C)** Western blot analysis of the PI3K and AKT pathways in γδT cells from tumors with different dose of VEGFR2-TKI therapy (γδT cells were sorted from 4 tumors as one donor). **(D)** Flow cytometry analysis of intracellular IFN-γ and IL17A expression of γδT cells derived from naive spleens treated with different doses of VEGFR2-TKI *in vitro*. **(E)** Western blot analysis of PI3K and AKT pathways in γδT cells derived from naive spleens treated with different doses of VEGFR2-TKI *in vitro* (γδT cells were sorted from 9 naive spleens as one donor). **(F)** Representative size of primary tumor after different dose VEGFR2-TKI therapy combined with PI3K agonist or inhibitor *in vivo* (n=5). **(G)** Intracellular IL17A expression of γδT cells from tumors after different dose VEGFR2-TKI therapy combined with PI3K agonist or inhibitor *in vivo* (n=5). Data are presented as the means ± SD from one representative experiment. Similar results were obtained from three independent experiments, n=4 mice each group, unless indicated otherwise. Statistical analysis was performed by one-way ANOVA. ns, not significant, *p < 0.05, **p < 0.01, and ***p < 0.001. See also **Supplementary Figure S3**.

VEGFR is divided into R1, R2 and R3. We found that VEGFR2-TKI therapy effectively reduced the expression of VEGFR2 on γδ T cells (**Supplementary Figure S3A** **)**, while high dose treatment increased the VEGFR1 expression without affecting VEGFR3 expression (**Figure 3A** and **Supplementary Figure S3B**). Furthermore, with the increase of VEGFR2-TKI concentration, the VEGFR1 expression of naive γδ T cells gradually increasing *in vitro* (**Figure 3B**), while VEGFR2 expression was opposite (**Supplementary Figure S3C**). Previous studies demonstrated that the PI3K signaling pathway can be activated by VEGFR2 or VEGFR1 (26, 27). We found that the PI3K-AKT pathway was significantly inhibited in γδ T cells sorted from tumors of the low-dose VEGFR2-TKI treatment group. On the contrary, γδ T cells from the high-dose and control groups had activated PI3K-AKT pathway (**Figure 3C** and **Supplementary Figure S3D**). In addition, we treated naive γδ T cells with VEGFR2-TKI *in vitro* and found progressively increased IL17A-secreting γδ T cells and decreased IFN-γ-producing γδ T cells with increasing drug concentration gradients (**Figure 3D**). Published studies discovered that activation of the PI3K-AKT pathway effectively promotes the secretion of IL17A (28, 29). Therefore, we speculated that the exchange of γδ T cell subtypes with different doses was attributed to the activation status of the PI3K-AKT pathway. We found that PI3K-AKT was also gradually activated in a dose-dependent manner of VEGFR2-TKI treatment (**Figure 3E** and **Supplementary Figure S3E**). Thus, we hypothesized that IL17A-producing γδ T cells are maintained by activated VEGFR2-PI3K-AKT signaling in the absence of treatment. However, activation of the VEGFR1-PI3K-AKT-IL17A pathway and the subsequent increased IL17A-producing γδ T cells accounts for the resistance to high-dose VEGFR2-TKI. To further prove the hypothesis, we designed *in vivo* experiments in which the PI3K-AKT agonist YS-49 was given to the low-dose group and the PI3K-AKT inhibitor Copanlisib was given to the high-dose group (**Supplementary Figure S3F**). We found that low-doses VEGFR2-TKI with YS-49 increased tumor size slightly, while the tumor from high-dose with Copanlisib group was significantly reduced, indicating that inhibiting the PI3K-AKT signaling pathway could reverse tumor resistance induced by high-dose treatment (**Figure 3F**). Further detection of tumor infiltrating γδT cells showed that low dose VEGFR2-TKI with YS-49 increased IL17A secretion, while high-dose VEGFR2-TKI with Copanlisib had the opposite effect (**Figure 3G**). PI3K signaling pathway is crucial for tumor cell (30) and tumor microenvironments (31–33). In order to further illustrate the role of PI3K on γδT cells, *in vitro* PI3K agonist treatment on naive γδT cells was operated and found that IL17A secreting increased gradually in accordance with PI3K agonist concentration (**Supplementary Figure S3G**). These results further confirmed the possibility of γδT cells-PI3K-IL17A pathway.

### High-Dose VEGFR2-TKI Therapy Facilitates “N2-Like” Neutrophil Differentiation Induced by IL17A-Producing γδ T Cells

**Figure 4:**
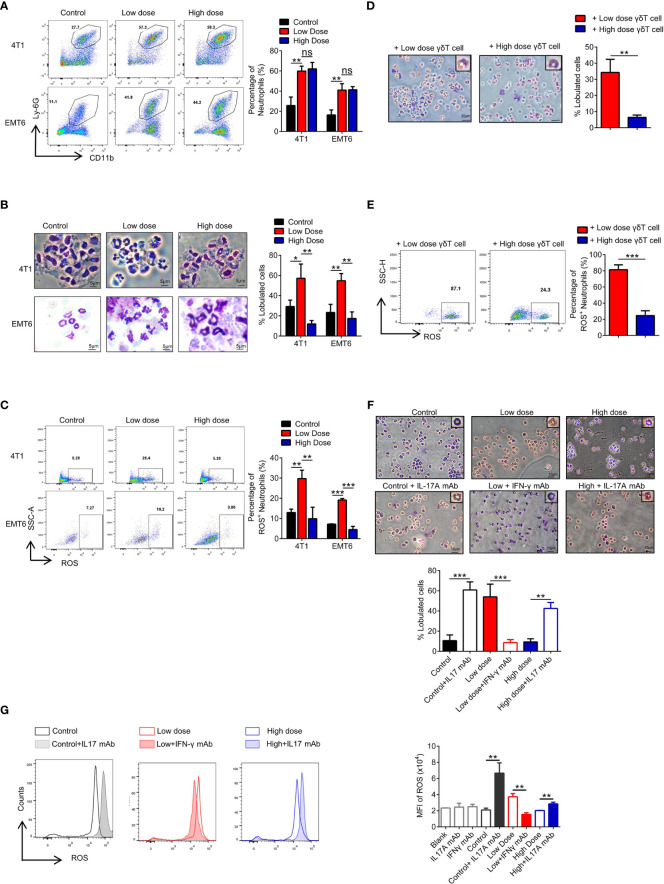
High-dose VEGFR2-TKI therapy facilitates N2-like neutrophils by IL17A-producing γδT cells. **(A)** Flow cytometry analysis of the percentage of neutrophils (CD45^+^CD11b^+^Ly-6G^+^) in the tumor after VEGFR2-TKI therapy. **(B)** Giemsa-stained neutrophils from the tumor after VEGFR2-TKI therapy *in vivo*. Bar=5 μm. **(C)** Reactive oxygen species (ROS) production of neutrophils in the tumor after VEGFR2-TKI therapy *in vivo*. **(D, E)** Representative images of Giemsa nuclear morphology analysis **(D)** and Frequency of ROS expression **(E)** of naive bone marrow (BM)-derived neutrophils, which were co-cultured with γδT cells sorted from tumors after different dose of VEGFR2-TKI therapy. Bar=20 μm. **(F, G)** Representative images of Giemsa nuclear morphology analysis **(F)** and MFI of ROS expression by flow cytometry analysis **(G)** in BM-derived neutrophils co-cultured with different tumor culture supernatants after VEGFR2-TKI therapy and (or) IL17 mAb or IFN-γ mAb added *in vitro*. Bar=10 μm. Data are presented as the means ± SD from one representative experiment. Similar results were obtained from three independent experiments, n=4 mice each group, unless indicated otherwise. Statistical analysis was performed by two-tailed unpaired Student’s t test **(A–E)** and one-way ANOVA **(F, G)**. ns, not significant, *p < 0.05, **p < 0.01, and ***p < 0.001. See also **Supplementary Figure S4**.

Previous studies have shown that both IFN-γ and IL17A have effects on the development of neutrophils (34), and we found that neutrophil numbers were significantly increased after VEGFR2-TKI treatment regardless of the therapeutic dose (**Figure 4A**). Fridlender (35) reported that neutrophils can be classified into an antitumorigenic type (called the ‘‘N1 phenotype’’) and a protumorigenic type (the ‘‘N2 phenotype’’). To assess the morphology of tumor-associated neutrophils, intratumoral CD45^+^CD11b^+^Ly6G^+^Ly6C^-^ cells were isolated. We observed that neutrophils in the low-dose group were more lobulated and hypersegmented. While in the high-dose group, neutrophils appeared to have characteristically circular nuclei (immature morphology) (**Figure 4B**). Production of reactive oxygen species (ROS), the key anti-tumor product of N1 neutrophils, was higher in neutrophils of the low-dose group than in those of the high-dose group (**Figure 4C**). Furthermore, we treated neutrophils with different doses of VEGFR2-TKI *in vitro* and found that the nucleus segmentation and ROS production of the neutrophils were not affected by the drug concentration (**Supplementary Figures S4A, B**). We also isolated γδ T cells from tumors treated with low-dose or high-dose VEGFR2-TKI and then cocultured them indirectly with naive neutrophils in a transwell chamber. The γδ T cells from low dose group could effectively promote the maturation of neutrophils and the secretion of ROS, while the γδ T cells from high-dose group did not have this ability (**Figures 4D, E** and **Supplementary Figure S4C**). To further demonstrate that neutrophils differ depending on the cytokines secreted in the tumor, we extracted culture supernatants from tumor tissues after treatment with different doses for induction of naive neutrophils, and an anti-IL17A mAb and anti-IFN-γ mAb were given as appropriate. The results showed that the anti-IL17A mAb could inhibit N2-phenotype induction in neutrophils mediated by high-dose tumor culture supernatant, while the anti-IFN-γ mAb could induce the transformation of neutrophils from the N1 phenotype to the N2 phenotype (**Figures 4F, G** and **Supplementary Figure S4D**). The above results illustrate that low doses of VEGFR2-TKI induce γδ T cells to secrete IFN-γ to promote neutrophil differentiation into the N1 phenotype, while at high-dose VEGFR2-TKI treatment, γδ T cells secrete IL17A to promote neutrophil differentiation into the N2 phenotype.

### High-Dose VEGFR2-TKI Therapy Exhausts CD8^+^ T Cells in Tumors

**Figure 5:**
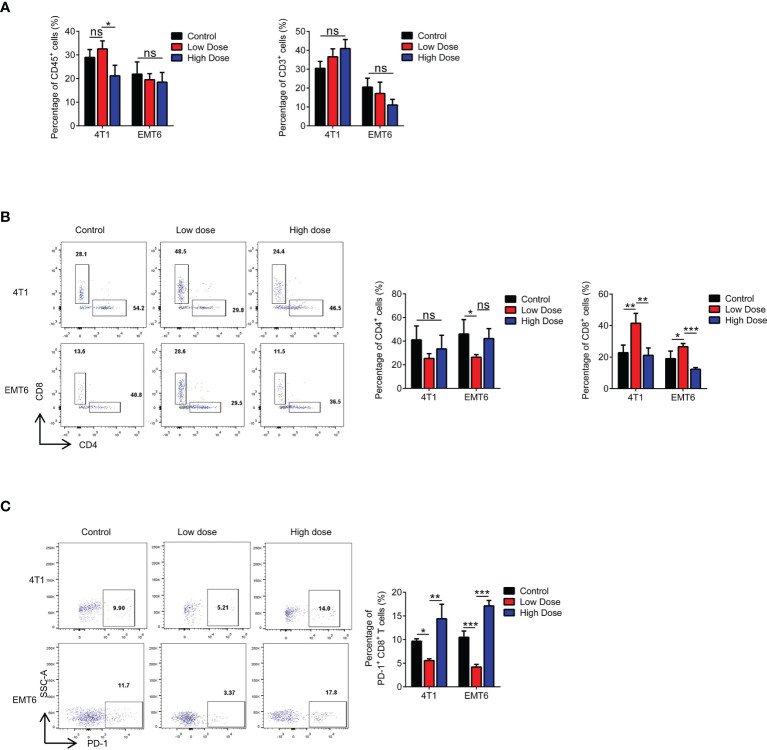
High-dose VEGFR2-TKI therapy exhausts CD8^+^ T cells in the tumor. **(A)** Flow cytometry analysis of immune cells (CD45^+^) and T cells (CD45^+^CD3^+^) in the tumor after VEGFR2-TKI therapy. **(B)** Frequency of CD4^+^ T cells (CD45^+^CD3^+^CD4^+^) and CD8^+^ T cells (CD45^+^CD3^+^CD8^+^) in the tumor. **(C)** Frequency of PD-1 expression on CD8^+^ T cells in the tumor after VEGFR2-TKI therapy. Data are presented as the means ± SD from one representative experiment. Similar results were obtained from three independent experiments, n=4 mice each group, unless indicated otherwise. Statistical analysis was performed by two-tailed unpaired Student’s t test, ns, not significant, *p < 0.05, **p < 0.01, and ***p < 0.001. See also **Supplementary Figure S5**.

To further clarify the proposed “γδ T cells- IL17A- N2 neutrophils- immunosuppression” hypothesis, we examined functional changes in T cells in the tumor microenvironment. The proportion of CD3^+^ T cells was independent of the therapeutic dose (**Figure 5A**). However, the CD4/CD8 ratio changed markedly, which was reflected by a significant decrease in the number of CD8^+^ T cells after high-dose treatment (**Figure 5B**). Further tests showed that PD-1 expression was increased on CD8^+^ T cells after high-dose treatment (**Figure 5C**). We also detected PD-L1 expression on tumor cells and found that it did not change with the different VEGFR2-TKI doses (**Supplementary Figures S5A, B**), indicating that the PD-1 expression on CD8^+^ T cells was upregulated for other reasons.

### Exhaustion of CD8^+^ T Cells Is Attributed to Neutrophils After High-Dose VEGFR2-TKI Therapy

**Figure 6:**
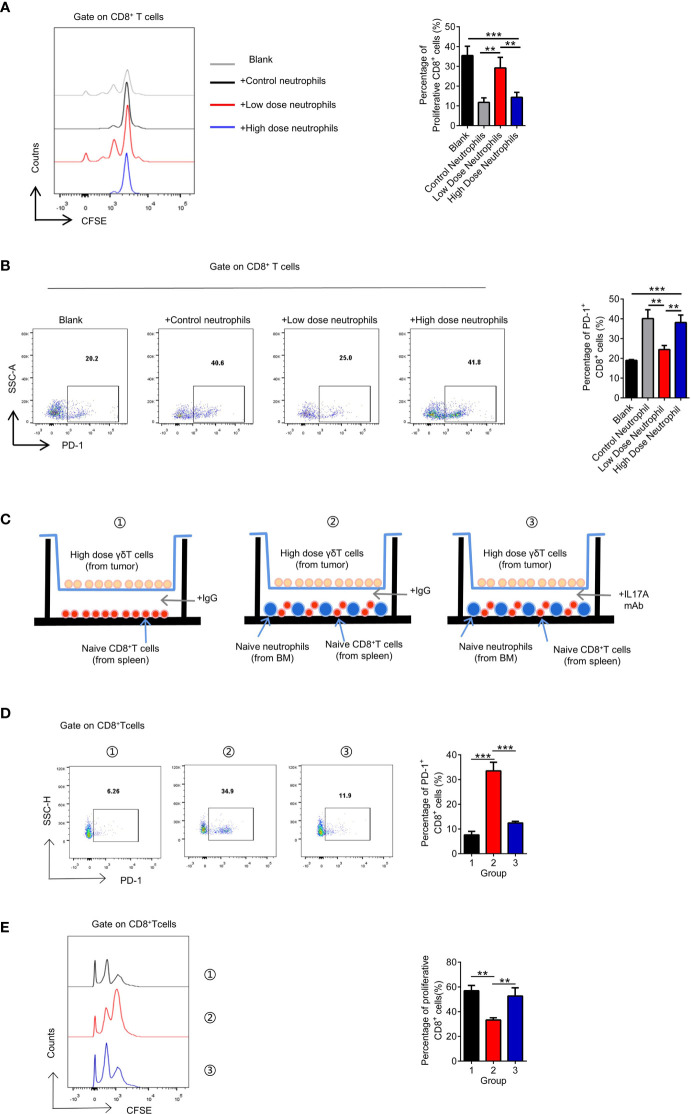
The exhaustion of CD8^+^ T cells is attributed to neutrophils after high-dose VEGFR2-TKI therapy. **(A, B)** Quantification of proliferation of CD8^+^ T cells (CFSE^low^CD8^+^) **(A)** and PD-1 expression on CD8^+^ T cells **(B)** after co-culture with different groups of neutrophils sorted from the tumor after VEGFR2-TKI therapy. CD8^+^ T cells were derived from naive spleens. **(C)** Schematic illustration of CD8^+^ T cells and neutrophils indirect co-cultrured with high dose of γδT cells sorted from the tumor after VEGFR2-TKI therapy shown in 6D and 6E. CD8^+^ T cells were derived from naive spleens. Neutrophils were derived from bone marrow. Transwell chamber=0.4μm. **(D, E)** Quantification of PD-1 expression on CD8^+^ T cells **(D)** and proliferation of CD8^+^ T cells (CFSE^low^CD8^+^) **(E)** after co-cultured with neutrophils and high dose of γδT cells sorted from the tumor after VEGFR2-TKI therapy shown in 6C. Data are presented as the means ± SD from one representative experiment. Similar results were obtained from three independent experiments, n=4 mice each group, unless indicated otherwise. Statistical analysis was performed by one-way ANOVA. **p < 0.01, and ***p < 0.001. See also **Supplementary Figure S6**.

It has been suggested that N2 neutrophils play an immunosuppressive role in the tumor microenvironment (36). Therefore, we examined the key molecules reported to play immunosuppressive function of N2 neutrophils, including Arginase, NO and PGE2, and found that PGE2 significantly increased in the high-dose treatment group, which may be the mechanism of immunosuppressive function (**Supplementary Figure S6A**). We cocultured neutrophils from tumors treated with different doses of VEGFR2-TKI with naive CD8^+^ T cells and found that only the neutrophils from the high-dose treatment group had immunosuppressive effects and significantly upregulated PD-1 expression on the CD8^+^ T cells (**Supplementary Figures S6A, B**). Furthermore, changes in the proportion and function of CD8^+^ T cells were not affected by γδ T cells in tumors treated with different therapeutic doses (**Supplementary Figures S6B, C**) or by the dose of VEGFR2-TKI (**Supplementary Figures S6D, E**). Therefore, we hypothesized that the drug resistance observed with high-dose VEGFR2-TKI treatment was due to the secretion of IL17A by γδ T cells, polarizing neutrophils into the N2 phenotype. N2 neutrophils play an immunosuppressive role, which reduces the number of CD8^+^ T cells and increases the expression level of PD-1, leading to the progression of breast cancer. In order to confirm the hypothesis, we designed co-culture system *in vitro* (**Supplementary Figure S6C**). We found that tumor-infiltrating γδT cells in the high-dose treatment group can only exert immunosuppressive effects in the presence of neutrophils, and this effect can be antagonized by IL17A mAb (**Figures 6D, E**).

### Combination With a mAb Can Restore the Efficacy of High-Dose VEGFR2-TKI Therapy

**Figure 7:**
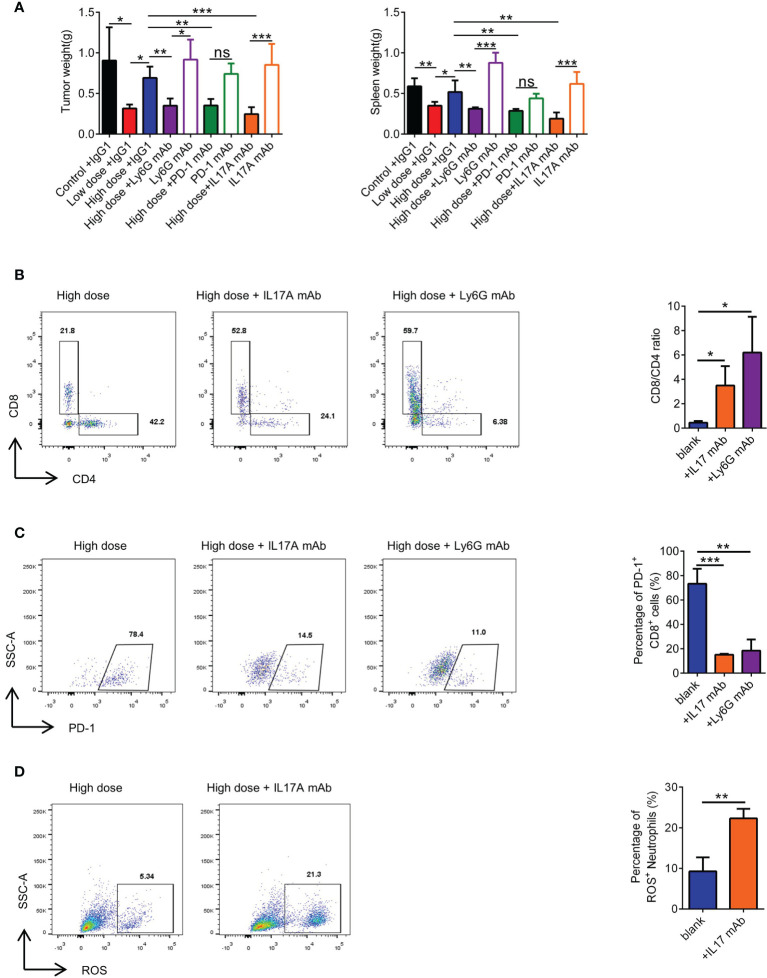
Combination with immunotherapeutic monoclonal antibody can rescue high-dose VEGFR2-TKI therapy. **(A)** Representative weight of primary tumor and spleen after high-dose VEGFR2-TKI therapy and(or) IL17A mAb, PD-1 mAb or Ly6G mAb. **(B)** Frequency of CD4^+^ T cells and CD8^+^ T cells in the tumor after high-dose VEGFR2-TKI therapy and(or) IL17A mAb or Ly6G mAb. **(C)** Frequency of PD-1 expression on CD8^+^ T cells in the tumor after high-dose VEGFR2-TKI therapy and(or) IL17A mAb or Ly6G mAb. **(D)** The expression of ROS in neutrophils after high-dose VEGFR2-TKI therapy and (or) IL17A mAb. Data are presented as the means ± SD from one representative experiment. Similar results were obtained from three independent experiments, n=5 mice each group, unless indicated otherwise. Statistical analysis was performed by two-tailed unpaired Student’s t test **(D)** and one-way ANOVA **(A–C)**. ns, not significant, *p < 0.05, **p < 0.01, and ***p < 0.001. See also **Supplementary Figure S7**.

To corroborate our pathway hypothesis, in 4T1 orthotopic breast cancer mice model, VEGFR2-TKI was treated alone or in combination with multiple immunotherapy, including an anti-IL17A mAb, anti-PD-1 mAb and anti-Ly6G mAb. Anti-Ly6G mAb can remove neutrophils from mouse body (**Supplementary Figure S7A**). We found that mAb combination was effective, especially in the anti-IL17A mAb group (**Figure 7A** and **Supplementary Figure S7B**). Notably, application of the anti-IL17A mAb, anti-PD-1 mAb or anti-Ly6G mAb alone may not be sufficient for tumor growth inhibition. What’s more, VEGFR2-TKI in combination with immunotherapy was seen to reduce lung metastasis (**Supplementary Figure S7C**), although it cannot be ruled out that it is affected by the reduction of primary tumor burden. More importantly, VEGFR2-TKI combined with the anti-IL17A mAb and anti-Ly6G mAb restored the percentage of CD8^+^ T cells and effectively reduced the expression of PD-1 (**Figures 7B, C**). In addition, the anti-IL17A mAb restored the ROS production of neutrophils in the tumor (**Figure 7D**). In general, high-dose of VEGFR2-TKI therapy combined with immunotherapy can relieve tumor drug resistance induced by high-dose therapy and is closely associated with γδT cells-IL17-neutrophils.

## Discussion

**Figure 8:**
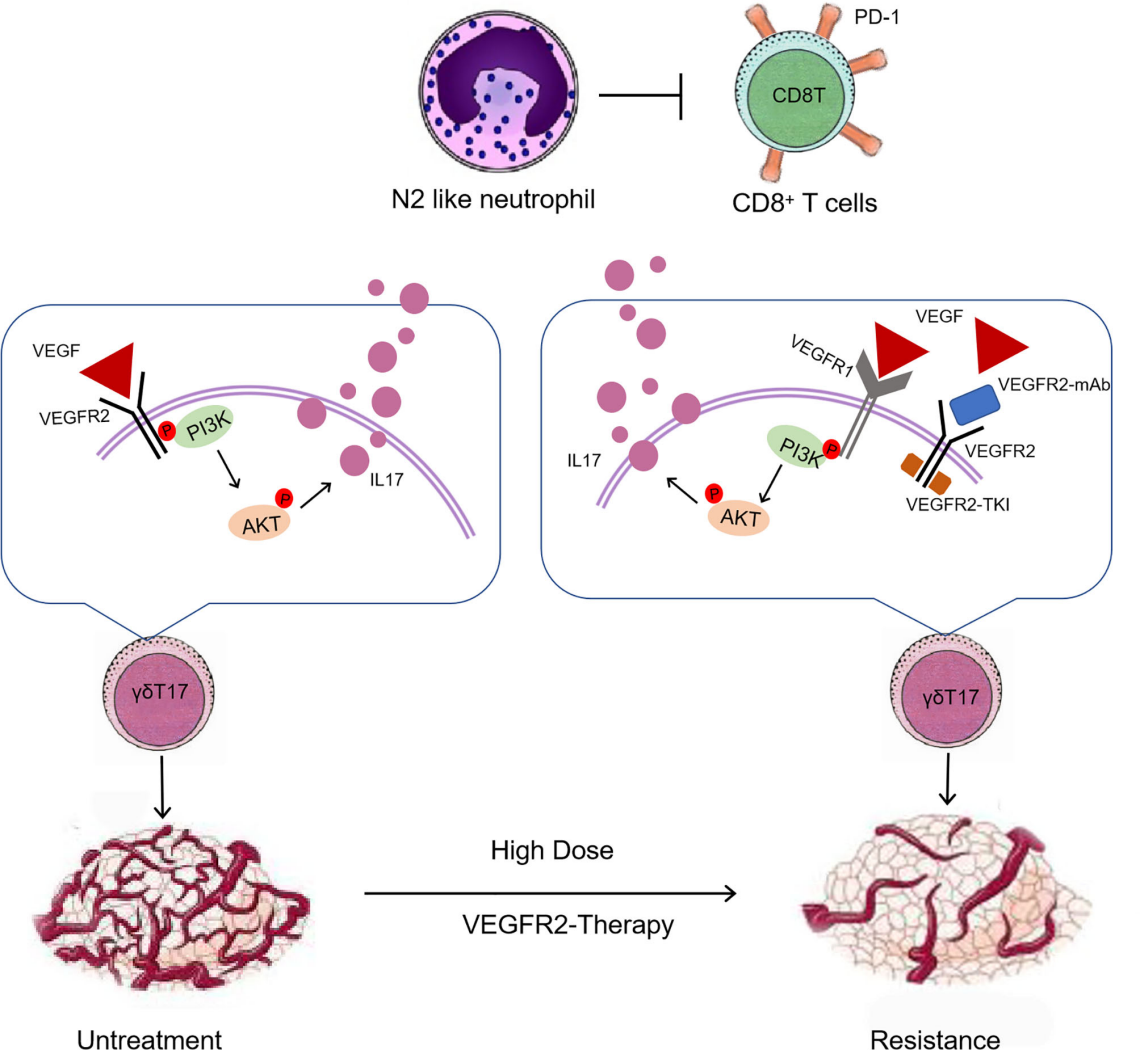
An overview schematic demonstrating that high-dose anti-VEGFR2 treatment modifies the tumor immunosuppression microenvironment *via* “γδT cell-IL17A-neutrophil” axis.

In orthotopic breast cancer models of 4T1 and EMT6 and the MMTV-PyMT model of spontaneous breast cancer, high-dose anti-VEGFR2 treatment was found to cause resistance to VEGFR2 monoclonal antibody and VEGFR2-TKI. We determined that intratumoral γδ T cells and neutrophils are involved in driving responsiveness and resistance to antiangiogenic therapy with a VEGFR2-TKI therapy. High-dose VEGFR2-TKI treatment induced IL17A production by tumor-infiltrating γδ T cells through the VEGFR1-PI3K-AKT pathway, while IL17A promoted “N2” neutrophil polarization, driving immunosuppression and conferring resistance to anti-VEGFR2 treatment (**Figure 8**).

VEGFR2 is widely expressed in blood vessels, especially tumor microvessels. Furthermore, VEGFR2 has been detected on various types of immune cells, such as macrophages, T cells and dendritic cells (37). It is an effective therapeutic target for tumor angiogenesis, but drug resistance caused by high-dose treatment is the current limitation of clinical treatment. Our study found that VEGFR2 expression was higher in T cells than in myeloid cells. Previous studies revealed that regulatory T cells (Tregs) are the most common VEGFR2-expressing T cells (38). However, T cells can be classified by T cell receptors (TCRs) into structural subsets, including αβ and γδ T cells (39). In the mouse breast cancer model, γδ T cells exhibited the highest VEGFR2 expression among all immune cells, implying that anti-VEGFR2 therapy may influence the function of γδ T cells. Nevertheless, the role of γδ T cells has not been studied in anti-VEGFR2 therapy resistance. Furthermore, we found that γδ T cells polarized into cytotoxic IFN-γ-producing subsets in “response” phases, while in “relapse” phases, γδ T cells polarized into suppressive IL17A-producing subsets. Further study found that, in “relapse” phases, mobilized VEGFR1 activated the PI3K-AKT pathway and then elicited IL17A secretion despite VEGFR2 inhibition. These results suggest that the PI3K-AKT pathway is likely to be the switch for conversion among γδ T cell subsets, and the upregulation of VEGFR1 in γδ T cells may exert major drug resistance in breast cancer.

Studies have reported that myeloid cells, including monocytes, macrophages and neutrophils, play a key role in the tumor microenvironment and promote tumor growth and metastasis (40). Myeloid cells promote tumor angiogenesis and are also involved in responsiveness and resistance to antiangiogenic therapy (25, 41). Anti-VEGF therapy facilitates Ly6C^lo^ monocyte infiltration *via* the CX3CL1-CX3CR1 pathway, and CX3CR1^+^Ly6C^lo^ monocytes create an immunosuppressive microenvironment that mediates resistance to antiangiogenic therapy (42). Previous study showed that anti- VEGFR2 treatment resistance is concerned with the accumulated myeloid-derived suppressor cells recruited by GM-CSF in ovarian cancer (43). Other studies have also found that macrophages actively contribute to resistance to antiangiogenic therapy in ovarian cancer (44). Considering quite different component of tumor microenvironment among diverse tumor types, and the role of neutrophils in conferring resistance to antiangiogenic therapy never been studied, the mechanism of antiangiogenic therapy resistance in breast cancer needs to be studied in depth.

Fridlender’s study verified that tumor-associated neutrophils are a heterogeneous set of immune cells, which can be classified as antitumorigenic (the “N1” phenotype) or protumorigenic (the “N2” phenotype) (35). N1 neutrophils exert antitumor activities by secreting more immunoactivating cytokines, producing more ROS and expressing lower levels of arginase, while N2 neutrophils play an immunosuppressive role. We found that neutrophils in “response” phases had a mature neutrophil-like morphology and “N1” function, while in “relapse” phases, neutrophils had an immature neutrophil-like morphology and “N2” function.

In the tumor microenvironment, immune cells undergo dramatic phenotypic changes induced by various stimuli and exhibit different functions (45). In our study, IL17A induced neutrophil N2 polarization, and nuclear morphological analysis revealed characteristics of immature neutrophils. A recent study demonstrated that IL17A-producing γδ T cells can polarize neutrophils into an immunosuppressive phenotype. Unfortunately, we did not find a specific surface marker for N1/N2 conversion from the anti-VEGFR2 therapy resistance models, as only morphological and immunosuppressive functions are involved. Further experiments, especially single-cell sequencing, are needed to focus on the effect of IL17A and IFN-γ on neutrophil phenotypes.

Our findings implied that immunosuppression, rather than angiogenesis, in the breast cancer microenvironment is the crucial mechanism conferring resistance to anti-VEGFR2 therapy exerted by γδT cells and neutrophils. Previous studies have shown that combining antiangiogenic therapy with immunotherapies has potential translational significance for cancer therapy (46). Moreover, most studies suggest low doses of anti-VEGFR2 therapy can induce vascular normalization and improve antitumor immunity, and improve immunotherapeutic efficacy (22, 47). But, according to the “therapy window” of vascular normalization, the low dose and treatment duration is difficult in clinical practice (46). Indeed, the therapeutic dose of anti-VEGFR2 drug currently applied in the clinic is often considered as a full or high dose. The clinical trials, like KEYNOTE-426 and IMbrave 150, found that full or high dose anti-VEGFR2 therapy plus PD-1 inhibitor significantly longer survival in some tumor types, which implied that high dose anti-VEGFR2 therapy closely related to the inhibitory immune microenvironment (48, 49). Our data also provide compelling evidence for optimized therapeutic strategies, when high-dose anti-VEGFR2 monotherapy evolve resistance. By remodeling inhibitory immune microenvironment via targeting the multiple points of “γδT cell-IL17A-neutrophil” axis (such as an anti-IL17 mAb), which can resensitize resistant tumors to antiangiogenic therapy and generate relatively durable effects. The mechanism of resistance caused by changes in the immune microenvironment provided by our research provides a possible solution for resistance to anti-vascular therapy, that is, while paying attention to the anti-vascular effect, the immune microenvironment should also be paid attention to, besides, combining with high-dose anti-vascular therapy and immunotherapy may provide new strategy for therapy in breast cancer treatment.

## Conclusion

Our study provides a novel rationale for the immunomodulatory effects involved in anti-VEGFR2 therapy, like VEGFR2-TKI antiangiogenic therapy. VEGFR2-TKI can directly act on γδ T cells and increase the inhibitory effects of N2-like neutrophils on T cell function in the tumor microenvironment, providing potential antitumor strategies in aggressive breast cancer. It will be important to perform clinical trials to test the usefulness of anti-VEGFR2 therapy combined with immunotherapy.

## Data Availability Statement

The original contributions presented in the study are included in the article/**Supplementary Material**. Further inquiries can be directed to the corresponding authors.

## Ethics Statement

The animal study was reviewed and approved by the Ethics Committee of the Second Affiliated Hospital of Zhejiang University School of Medicine in accordance with the Declaration of Helsinki. Written informed consent was obtained from the owners for the participation of their animals in this study.

## Author Contributions

Conception and design, JH and ZW. Development of methodology, ZZ and CY. Acquisition of data (provided animals, acquired and managed patients, provided facilities, etc.), ZZ and CY. Analysis and interpretation of data (e.g., statistical analysis, biostatistics, computational analysis), ZZ, CY, and LZ. Writing, review, and/or revision of the manuscript, ZZ and LL. Administrative, technical, or material support (i.e., reporting or organizing data, constructing databases), YZ and KS. Study supervision, ZW. All authors contributed to the article and approved the submitted version.

## Funding

This work was supported by grants from the National Natural Science Foundation of China (81930079, 81872317, 81902981, 81902626) and Health Commission of Zhejiang Province (WKJ-ZJ-1803).

## Conflict of Interest

The authors declare that the research was conducted in the absence of any commercial or financial relationships that could be construed as a potential conflict of interest.

## Publisher’s Note

All claims expressed in this article are solely those of the authors and do not necessarily represent those of their affiliated organizations, or those of the publisher, the editors and the reviewers. Any product that may be evaluated in this article, or claim that may be made by its manufacturer, is not guaranteed or endorsed by the publisher.

## Supplementary Material

Supplementary Figure S1 related to **Figure 1**. **(A)** Schematic illustration of VEGFR2-TKI and VEGFR2 mAb of different therapeutic doses in 4T1 and EMT6 breast cancer models. i.g., intragastric administration. i.p., intraperitoneal injection. **(B)** Immunofluorescence of CD31 on breast cancer tissue after anti-VEGFR2 therapy in EMT6 model. Bar=200 μm. Green, CD31; blue, DAPI. **(C)** Analysis weight of spleens after different doses of anti-VEGFR2 therapy in 4T1 and EMT6 models. **(D)** Tumor growth curve of 4T1 and EMT6 breast cancer tissue after anti-VEGFR2 therapy. Data are presented as the means ± SD from one representative experiment. Similar results were obtained from three independent experiments, n=4 mice each group, unless indicated otherwise. Statistical analysis was performed by one-way ANOVA **(C)** and repeated-measures ANOVA **(D)**. ns, not significant, *p<0.05, **p<0.01, and ***p<0.001.

Supplementary Figure S2 related to **Figure 1** and **Figure 2**. **(A, B)** Schematic illustration **(A)** and anatomy results **(B)** for anti-VEGFR2 treatment in the MMTV-PyMT breast cancer model. i.g., intragastric administration. i.p., intraperitoneal injection. **(C)** Flow cytometry gate strategy of tumor-infiltrating myeloid cell and lymphocyte detection. Data are presented as the means ± SD from one representative experiment. Similar results were obtained from three independent experiments, n=4 mice each group, unless indicated otherwise. Statistical analysis was performed by one-way ANOVA **(B)**. ns, not significant, *p<0.05, **p<0.01, and ***p<0.001.

Supplementary Figure S3 related to **Figure 3**. **(A, B)** Flow cytometry analysis of VEGFR2 **(A)** and VEGFR3 **(B)** expression of γδT cells (CD45^+^CD3^+^TCRγδ^+^) in tumors after different dose of VEGFR2-TKI therapies. **(C)** Flow cytometry analysis of VEGFR2 expression of γδT cells derived from naive spleens treated with different doses of VEGFR2-TKI *in vitro*. **(D)** Western blot analysis of PI3K/actin and AKT/actin in γδ T cells from tumors with different dose of VEGFR2-TKI therapies (γδT cells were sorted from 4 tumors as one donor). **(E)** Western blot analysis of PI3K/actin and AKT/atin in γδ T cells derived from naive spleens treated with different doses of VEGFR2-TKI *in vitro* (γδ T cells were sorted from 9 naive spleens as one donor). **(F)** Schematic illustration of VEGFR2-TKI of different therapeutic doses combined with YS-49 (PI3K agoist) and Copanlisib (PI3K inhibitor) in 4T1 breast cancer models. *i.g.*, intragastric administration. *i.p.*, intraperitoneal injection. Qd, once a day. Qod once every other one day. **(G)** Flow cytometry analysis of IL17 expression of γδT cells derived from naive spleens treated with different doses of YS-49 *in vitro*. Data are presented as the means ± SD from one representative experiment. Similar results were obtained from three independent experiments, n=4 mice each group, unless indicated otherwise. Statistical analysis was performed by one-way ANOVA. ns, not significant, *p<0.05, **p<0.01, and ***p<0.001.

Supplementary Figure S4 related to **Figure 4**. **(A)** Giemsa-stained naive BM-derived neutrophils treated with different concentrations of VEGFR2-TKI *in vitro*. **(B)** Flow cytometry analysis of ROS expression of neutrophils treated with different concentrations of VEGFR2-TKI *in vitro*. **(C)** Giemsa-stained naive BM-derived neutrophils without any treatment. **(D)** Giemsa-stained neutrophils treated with or without IL17A mAb or IFN-γ mAb. Data are presented as the means ± SD from one representative experiment. Similar results were obtained from three independent experiments, n=4 mice each group, unless indicated otherwise. Statistical analysis was performed by one-way ANOVA. ns, not significant, *p<0.05, **p<0.01, and ***p<0.001.

Supplementary Figure S5 related to **Figure 5**. **(A)** Flow cytometry gating strategy of tumor cells. **(B)** Frequency of PD-L1 expression on tumor cells (CD45^-^EpCAM^+^) in the tumor after VEGFR2-TKI therapy. Data are presented as the means ± SD from one representative experiment. Similar results were obtained from three independent experiments, n=4 mice each group, unless indicated otherwise. Statistical analysis was performed by one-way ANOVA. ns, not significant, *p<0.05, **p<0.01, and ***p<0.001.

Supplementary Figure S6 related to **Figure 6**. **(A)** Compared biochemical parameters associated with immunosuppressive characterization of neutrophils including arginase (Functional kit), NO (Content detection kit) and PGE2 (ELISA) in tumor-infiltrating neutrophils after different dose of VEGFR2-TKI therapies. **(B, C)** Flow cytometry analysis of proliferation **(B)** and PD-1 expression **(C)** of CD8^+^ T cells after co-culturing with the different group of γδT cells sorting from the tumor after VEGFR2-TKI therapy. CD8^+^ T cells were derived from naive spleen. **(D, E)** Flow cytometry analysis of proliferation of CD8^+^ T cells (CFSE^low^CD8^+^) **(D)** and PD-1 expression of CD8^+^ T cells **(E)** treated with different concentrations of VEGFR2-TKI *in vitro*. Data are presented as the means ± SD from one representative experiment. Similar results were obtained from three independent experiments, n=4 mice each group, unless indicated otherwise. Statistical analysis was performed by one-way ANOVA. ns, not significant, *p<0.05, **p<0.01, and ***p<0.001.

Supplementary Figure S7 related to **Figure 7**. **(A)** Flow cytometry analysis of neutrophils in the PB of the 4T1 model treated with Ly-6G mAb. **(B)** Representative of primary tumor after high-dose VEGFR2-TKI therapy and(or) IL17A mAb, PD-1 mAb or Ly6G mAb. **(C)** H&E staining and quantification of lung metastasis in the 4T1 model (Black arrow) after multiple treatments. Bar=1 mm. Data are presented as the means ± SD from one representative experiment. Similar results were obtained from three independent experiments, n=4 mice each group, unless indicated otherwise. Statistical analysis was performed by one-way ANOVA. ns, not significant, *p<0.05, **p<0.01, and ***p<0.001.
