# Supplementary figures and images for: “γδT Cell-IL17A-Neutrophil” Axis Drives Immunosuppression and Confers Breast Cancer Resistance to High-Dose Anti-VEGFR2 Therapy
Source: Front Immunol. 2021 Oct 15;12:699478. doi: 10.3389/fimmu.2021.699478 (PMC8554133; doi:10.3389/fimmu.2021.699478)

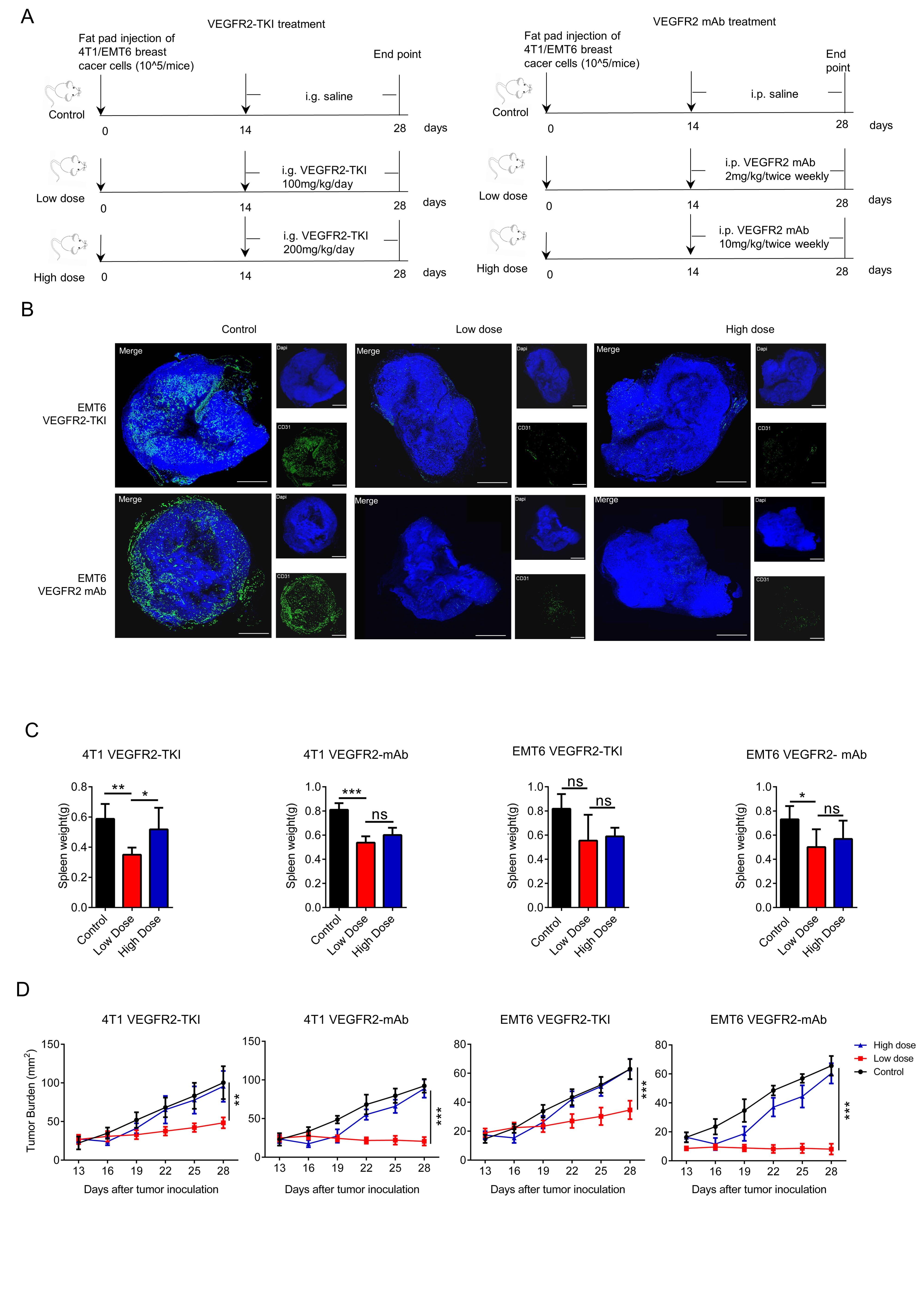

Supplement: Supplementary Figure S1 — related to Figure 1 . (A) Schematic illustration of VEGFR2-TKI and VEGFR2 mAb of different therapeutic doses in 4T1 and EMT6 breast cancer models. i.g., intragastric administration. i.p., intraperitoneal injection. (B) Immunofluorescence of CD31 on breast cancer tissue after anti-VEGFR2 therapy in EMT6 model. Bar=200 μm. Green, CD31; blue, DAPI. (C) Analysis weight of spleens after different doses of anti-VEGFR2 therapy in 4T1 and EMT6 models. (D) Tumor growth curve of 4T1 and EMT6 breast cancer tissue after anti-VEGFR2 therapy. Data are presented as the means ± SD from one representative experiment. Similar results were obtained from three independent experiments, n=4 mice each group, unless indicated otherwise. Statistical analysis was performed by one-way ANOVA (C) and repeated-measures ANOVA (D). ns, not significant, *p<0.05, **p<0.01, and ***p<0.001. [file Image_1.jpeg]

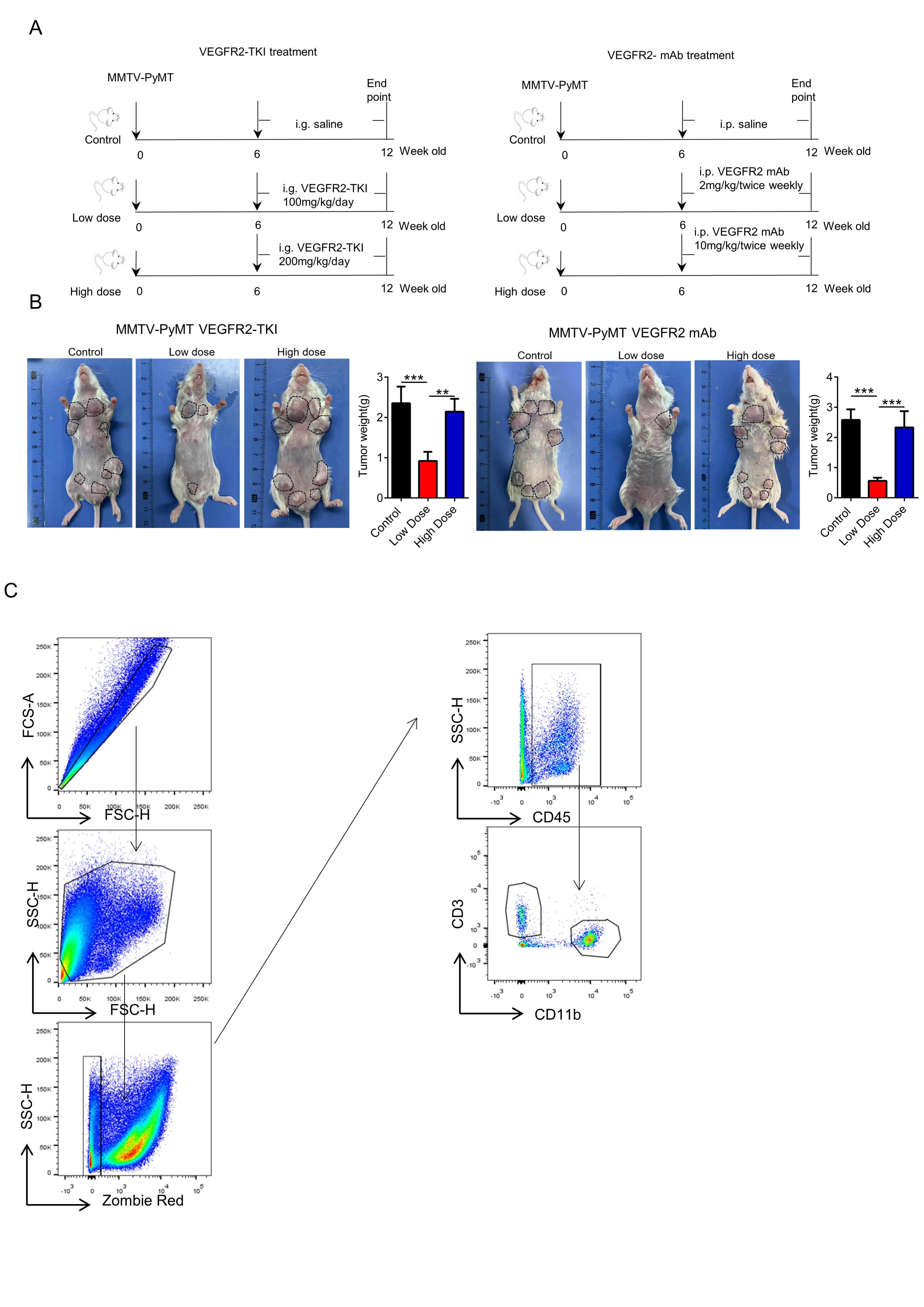

Supplement: Supplementary Figure S2 — related to Figure 1 and Figure 2 . (A, B) Schematic illustration (A) and anatomy results (B) for anti-VEGFR2 treatment in the MMTV-PyMT breast cancer model. i.g., intragastric administration. i.p., intraperitoneal injection. (C) Flow cytometry gate strategy of tumor-infiltrating myeloid cell and lymphocyte detection. Data are presented as the means ± SD from one representative experiment. Similar results were obtained from three independent experiments, n=4 mice each group, unless indicated otherwise. Statistical analysis was performed by one-way ANOVA (B). ns, not significant, *p<0.05, **p<0.01, and ***p<0.001. [file Image_2.jpeg]

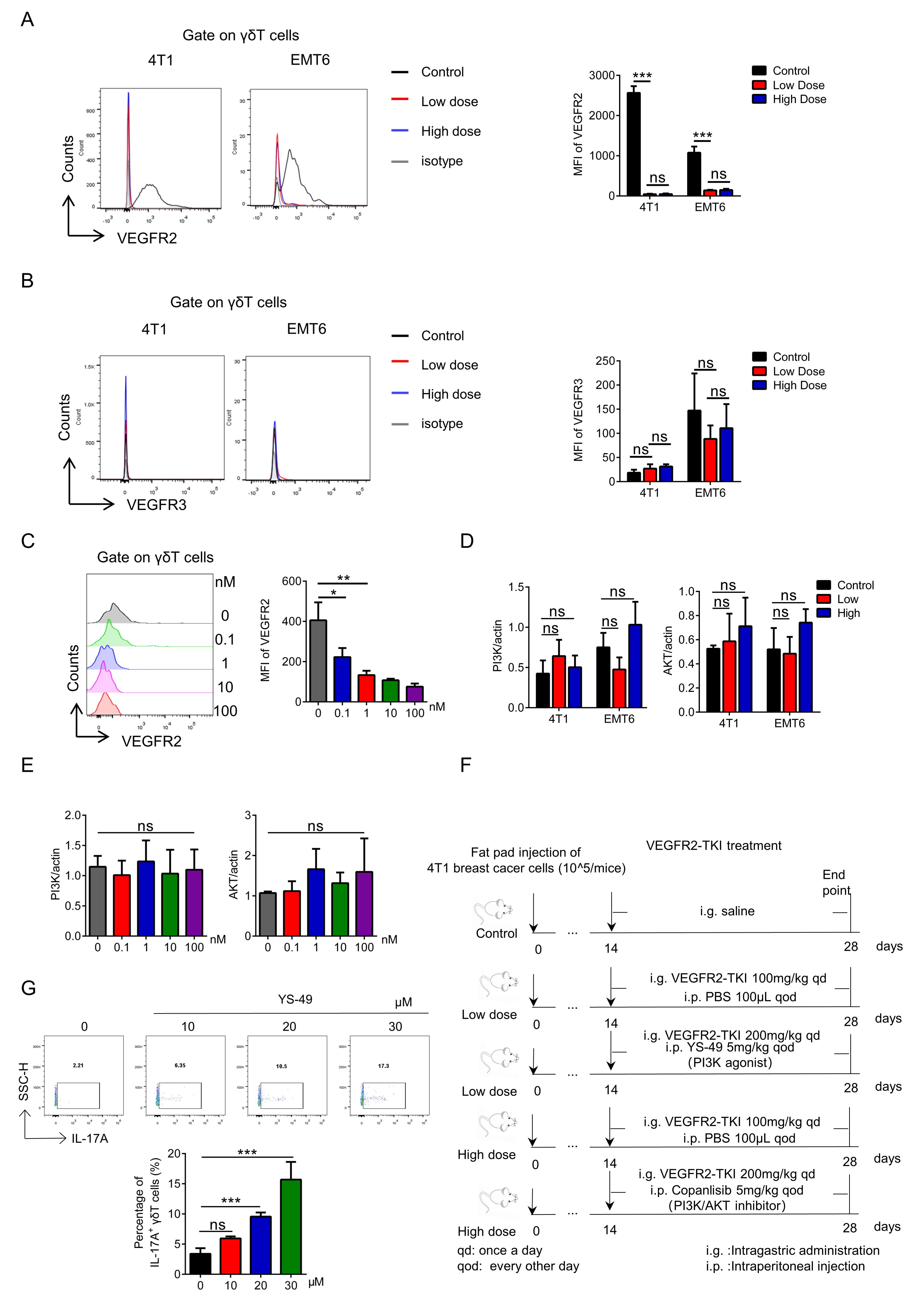

Supplement: Supplementary Figure S3 — related to Figure 3 . (A, B) Flow cytometry analysis of VEGFR2 (A) and VEGFR3 (B) expression of γδT cells (CD45+CD3+TCRγδ+) in tumors after different dose of VEGFR2-TKI therapies. (C) Flow cytometry analysis of VEGFR2 expression of γδT cells derived from naive spleens treated with different doses of VEGFR2-TKI in vitro. (D) Western blot analysis of PI3K/actin and AKT/actin in γδ T cells from tumors with different dose of VEGFR2-TKI therapies (γδT cells were sorted from 4 tumors as one donor). (E) Western blot analysis of PI3K/actin and AKT/atin in γδ T cells derived from naive spleens treated with different doses of VEGFR2-TKI in vitro (γδ T cells were sorted from 9 naive spleens as one donor). (F) Schematic illustration of VEGFR2-TKI of different therapeutic doses combined with YS-49 (PI3K agoist) and Copanlisib (PI3K inhibitor) in 4T1 breast cancer models. i.g., intragastric administration. i.p., intraperitoneal injection. Qd, once a day. Qod once every other one day. (G) Flow cytometry analysis of IL17 expression of γδT cells derived from naive spleens treated with different doses of YS-49 in vitro. Data are presented as the means ± SD from one representative experiment. Similar results were obtained from three independent experiments, n=4 mice each group, unless indicated otherwise. Statistical analysis was performed by one-way ANOVA. ns, not significant, *p<0.05, **p<0.01, and ***p<0.001. [file Image_3.jpeg]

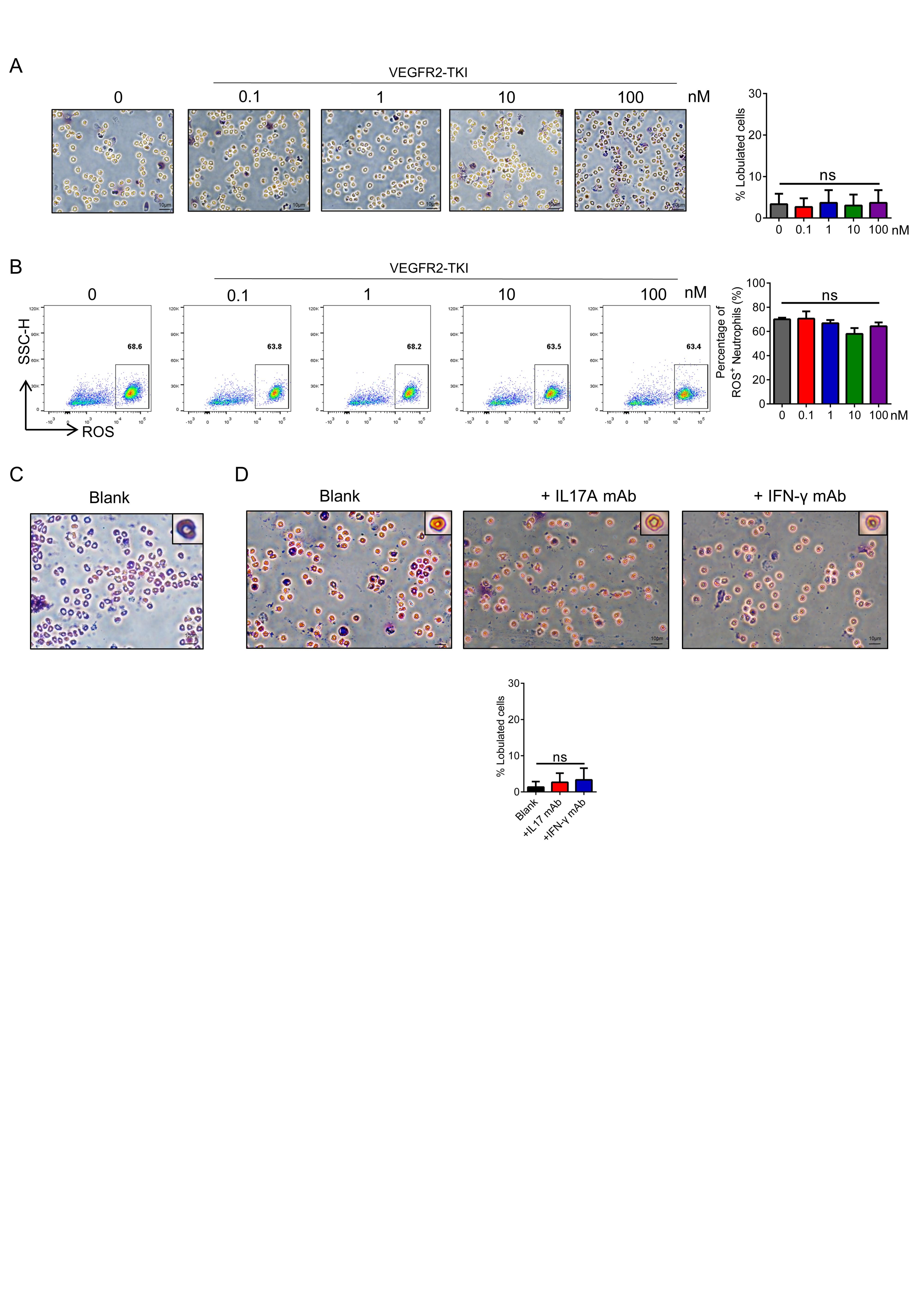

Supplement: Supplementary Figure S4 — related to Figure 4 . (A) Giemsa-stained naive BM-derived neutrophils treated with different concentrations of VEGFR2-TKI in vitro. (B) Flow cytometry analysis of ROS expression of neutrophils treated with different concentrations of VEGFR2-TKI in vitro. (C) Giemsa-stained naive BM-derived neutrophils without any treatment. (D) Giemsa-stained neutrophils treated with or without IL17A mAb or IFN-γ mAb. Data are presented as the means ± SD from one representative experiment. Similar results were obtained from three independent experiments, n=4 mice each group, unless indicated otherwise. Statistical analysis was performed by one-way ANOVA. ns, not significant, *p<0.05, **p<0.01, and ***p<0.001. [file Image_4.jpeg]

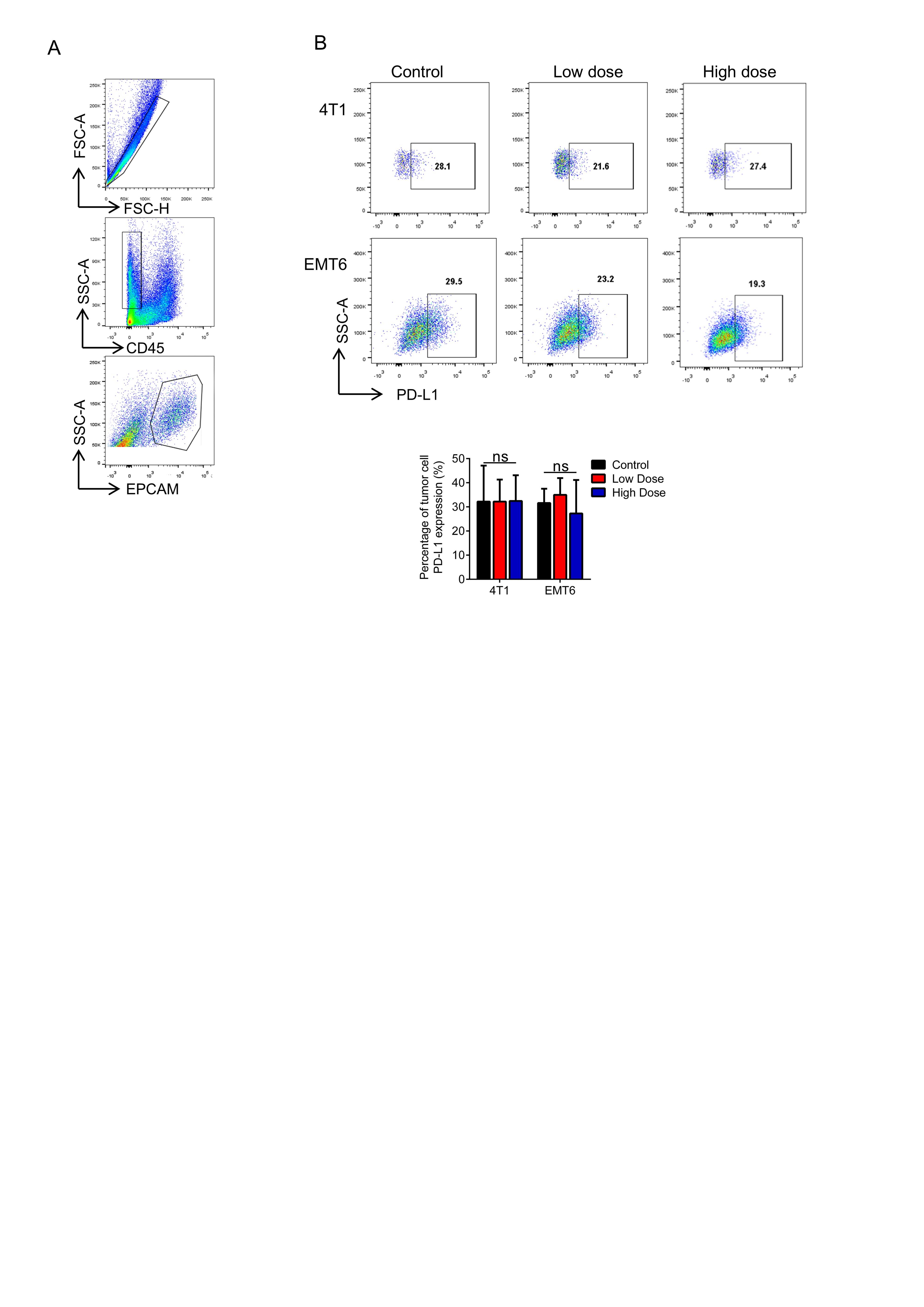

Supplement: Supplementary Figure S5 — related to Figure 5 . (A) Flow cytometry gating strategy of tumor cells. (B) Frequency of PD-L1 expression on tumor cells (CD45-EpCAM+) in the tumor after VEGFR2-TKI therapy. Data are presented as the means ± SD from one representative experiment. Similar results were obtained from three independent experiments, n=4 mice each group, unless indicated otherwise. Statistical analysis was performed by one-way ANOVA. ns, not significant, *p<0.05, **p<0.01, and ***p<0.001. [file Image_5.jpeg]

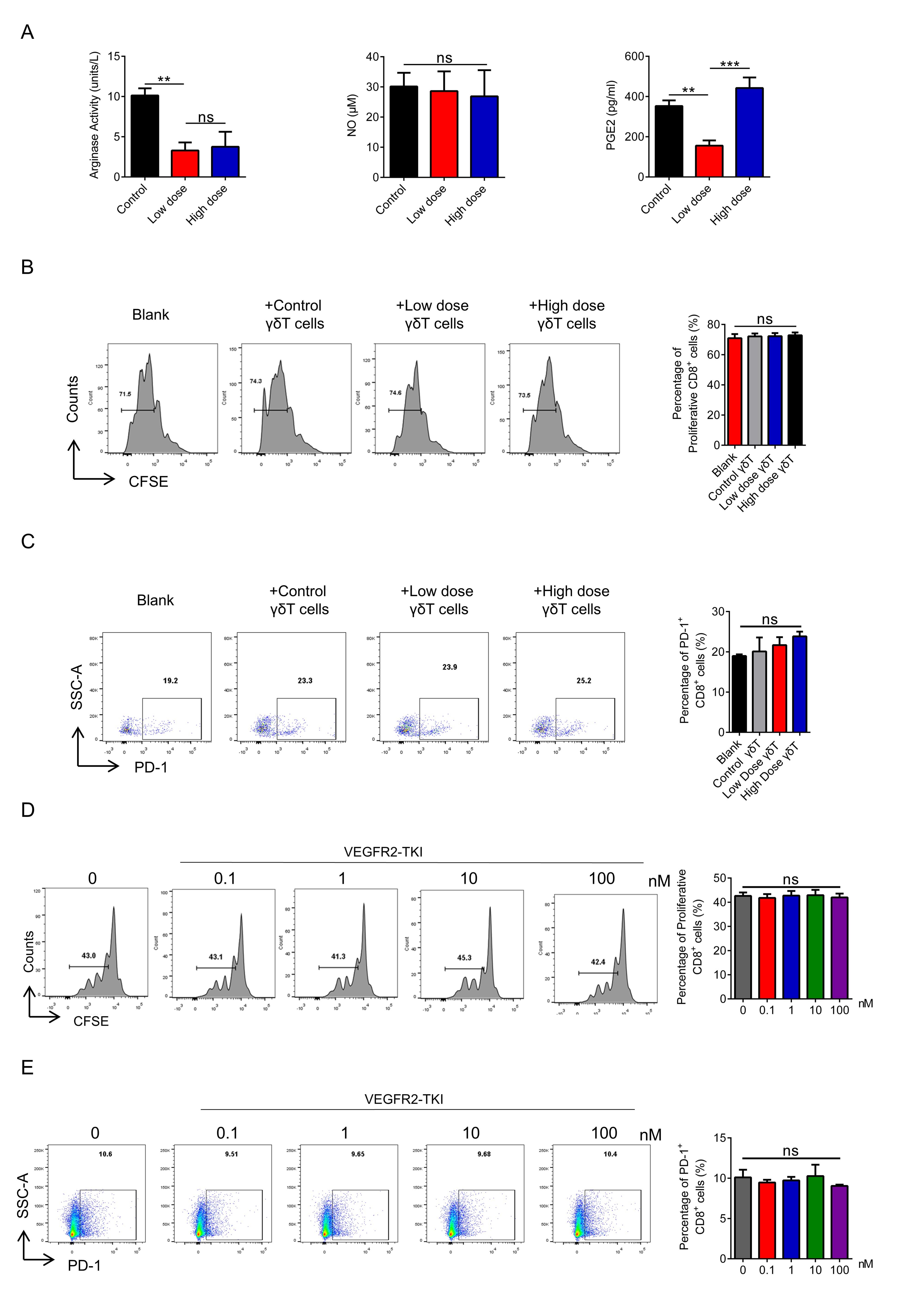

Supplement: Supplementary Figure S6 — related to Figure 6 . (A) Compared biochemical parameters associated with immunosuppressive characterization of neutrophils including arginase (Functional kit), NO (Content detection kit) and PGE2 (ELISA) in tumor-infiltrating neutrophils after different dose of VEGFR2-TKI therapies. (B, C) Flow cytometry analysis of proliferation (B) and PD-1 expression (C) of CD8+ T cells after co-culturing with the different group of γδT cells sorting from the tumor after VEGFR2-TKI therapy. CD8+ T cells were derived from naive spleen. (D, E) Flow cytometry analysis of proliferation of CD8+ T cells (CFSElowCD8+) (D) and PD-1 expression of CD8+ T cells (E) treated with different concentrations of VEGFR2-TKI in vitro. Data are presented as the means ± SD from one representative experiment. Similar results were obtained from three independent experiments, n=4 mice each group, unless indicated otherwise. Statistical analysis was performed by one-way ANOVA. ns, not significant, *p<0.05, **p<0.01, and ***p<0.001. [file Image_6.jpeg]

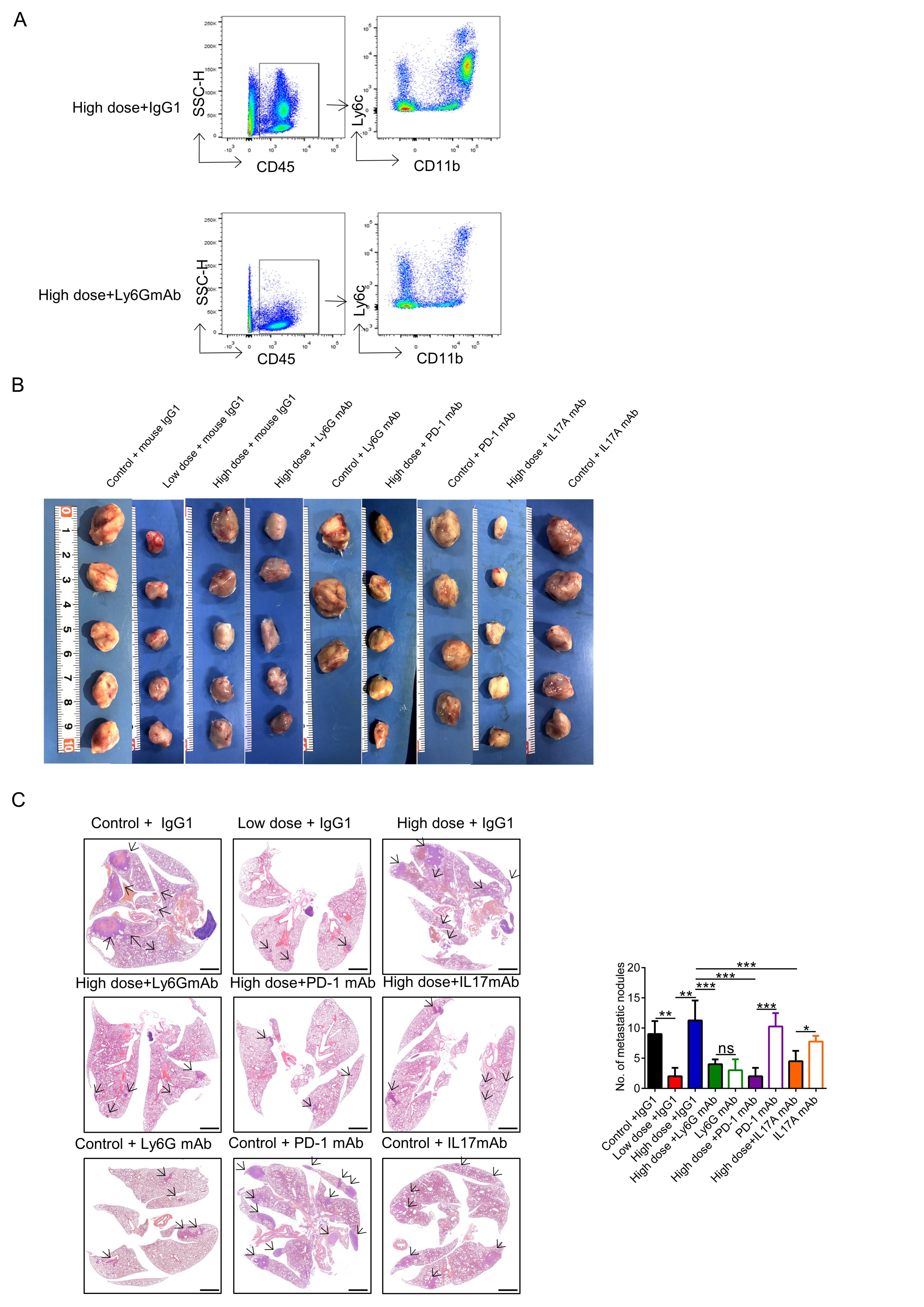

Supplement: Supplementary Figure S7 — related to Figure 7 . (A) Flow cytometry analysis of neutrophils in the PB of the 4T1 model treated with Ly-6G mAb. (B) Representative of primary tumor after high-dose VEGFR2-TKI therapy and(or) IL17A mAb, PD-1 mAb or Ly6G mAb. (C) H&E staining and quantification of lung metastasis in the 4T1 model (Black arrow) after multiple treatments. Bar=1 mm. Data are presented as the means ± SD from one representative experiment. Similar results were obtained from three independent experiments, n=4 mice each group, unless indicated otherwise. Statistical analysis was performed by one-way ANOVA. ns, not significant, *p<0.05, **p<0.01, and ***p<0.001. [file Image_7.jpeg]
